# Morphological characters in light of new molecular phylogenies: the caudal-fin skeleton of Ovalentaria

**DOI:** 10.1098/rsos.211605

**Published:** 2022-01-12

**Authors:** Philipp Thieme, Nalani K. Schnell, Kerryn Parkinson, Timo Moritz

**Affiliations:** ^1^ Deutsches Meeresmuseum, Katharinenberg 14-20, 18439 Stralsund, Germany; ^2^ Institute of Zoology and Evolutionary Research, Friedrich-Schiller-University Jena, Ebertstrasse 1, 07743 Jena, Germany; ^3^ Institut Systématique Evolution Biodiversité (ISYEB), Muséum national d'Histoire naturelle, CNRS, Sorbonne Université, EPHE, Station Marine de Concarneau, Place de la Croix, 29900 Concarneau, France; ^4^ Australian Museum Research Institute, Australian Museum, 1 William Street, Sydney, New South Wales 2010, Australia; ^5^ Institute of Biological Sciences, University of Rostock, Albert-Einstein-Strasse 3, 18059 Rostock, Germany

**Keywords:** Teleostei, comparative anatomy, ancestral character state reconstruction, Cichlidae, Atherinomorpha, locomotion

## Abstract

The Ovalentaria is a taxon of teleosts that has been proposed based on molecular analyses only. Previously widely separated families are assembled in this taxon. For the first time, the Ovalentaria are analysed using a comparative morphological approach. The caudal-fin skeleton of 355 species covering all 48 ovalentarian families are examined in cleared and stained specimens, µCT datasets and X-ray images as well as from the literature. A total of 38 morphological characters are evaluated and used for ancestral character state reconstructions and phylogenetic analyses. Results provide hypotheses for a scenario of the evolution of the caudal-fin skeleton and its ground plan in Ovalentaria. An evolutionary trend towards the reduction of skeletal elements in the caudal fin is observed. Connections between the evolution of the caudal-fin skeleton and modes of locomotion found in ovalentarian taxa are discussed. Phylogenetic analyses based on the caudal-fin morphology provide topologies for intra-ovalentarian relationships that largely agree with molecular hypotheses.

## 1. Introduction

Within the past two decades, our knowledge of the phylogenetic systematic of actinopterygians has been redefined by expansive molecular analyses [1–6]. Such studies provide us with phylogenies for all major actinopterygian clades. In the past, morphology was used to reconstruct phylogenetic relationships and the respective character evolution was discussed at the same time (e.g. [7–9]). Since the mid-2000s, mostly genetic data have been used to analyse phylogenetic relationships of actinopterygians and detailed discussions of morphological data have become rare. In many cases expansive molecular-based phylogenies propose relationships that have not been considered with morphological data and therewith stimulate new comparative studies to test those relationships (e.g. [6,10,11]). There are two advantages of using molecular analyses as a base for new comparative morphological studies: (i) systematic relationships retrieved from molecular data can provide a foundation for the reconstruction of the evolution of morphological structures and characters and (ii) phylogenetic hypotheses can be reviewed in the light of comparative morphological data. Many actinopterygian character complexes are suitable for such studies, e.g. the pectoral girdle, the median fins, the caudal fin, or a combination of such complexes.

**Figure 1.**
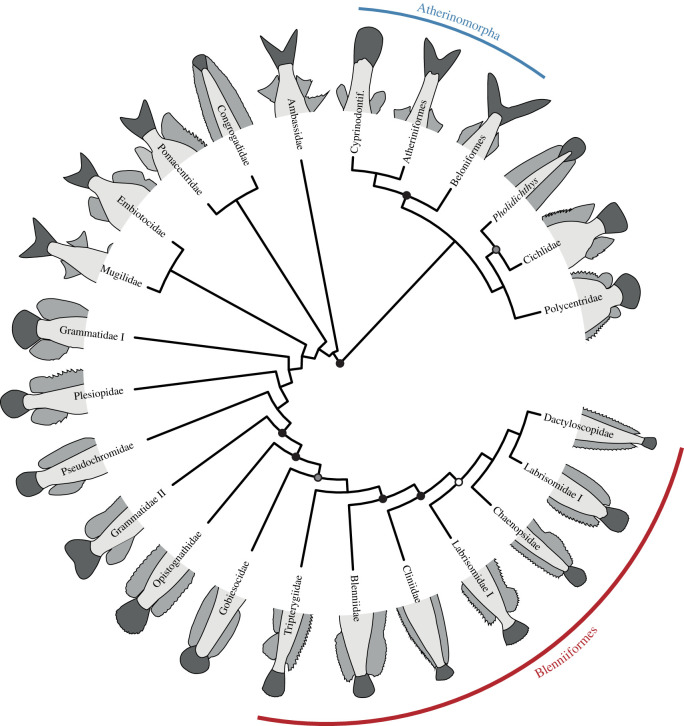
Phylogenetic tree modified from Betancur-R *et al*. [2] displaying all ovalentarian families and the typical caudal-fin shape of each family. Nodes with bootstrap support values above 80% are indicated by circles (black: 100–95%; grey: 94–90%; white: 89–80%).

Investigations of the caudal fin are a promising starting point as the evolution of this character complex had a major influence on diversity of teleost fishes [12,13]. This was promoted by the evolutionary transition from a polyural and heterocercal to a diural and homocercal caudal fin, both apomorphic characters of the Teleostei [14–16]. Diverse locomotory modes evolved [12,17,18], which is reflected by the many caudal-fin shapes (figure 1) [19], and these influenced the diversification of teleosts as a major taxon that contributes almost half of all vertebrate taxa [19–21]. Locomotion modes in teleosts comprise cruising and sprinting, accelerating and manoeuvring [22–24]. These are generally achieved by a combination of body and caudal-fin (BCF) propulsion and median and paired fin (MPF) propulsion. BCF propulsion is further subdivided, based on the length the propulsive waves travel through the fish body, in undulatory (whole body; e.g. anguilliform swimming), oscillatory (caudal peduncle; e.g. thunniform swimming) and intermediate modes (midway through body; e.g. carangiform swimming). Studies on the locomotion of fishes mostly focused on quantifying body movements, body kinematics, musculature employment and body and fin shapes [17,18,22–25]. While the shape of the caudal fin can give some information on the locomotion modes used, the caudal-fin skeleton may provide more detailed insight. However, information on the caudal-fin skeleton was rarely analysed in relation to modes of locomotion [26], although the caudal-fin skeleton was studied extensively for almost the past two centuries and new insights were constantly revealed [21,27–46]. This has resulted in the utilization of the morphological diversity of the caudal fin and its skeleton in phylogenetic analyses (e.g. [9,46,47–52]).

Combining the results of recent molecular phylogenetic studies with a morphological analysis of the caudal skeleton is a logical step to advance the knowledge of teleost evolutionary history. An exemplary taxon suited for such an approach is the Ovalentaria. This assemblage represents many taxa that previously were regarded to be distantly related within Percomorpha (*sensu* [53]), which is why there are no comparative morphological analyses of this taxon available. This presents an opportunity to evaluate caudal fins of these taxa in the light of a new phylogenetic hypothesis.

The taxon Ovalentaria was first proposed by Wainwright *et al.* [6] based on DNA sequence data from 10 nuclear loci. The taxon was retrieved again in subsequent analyses [1,2,54]. The Ovalentaria comprises 42 [19] to 48 [55] families. However, the monophyly of some of these families (e.g. Grammatidae, Labrisomidae) has been questioned [1,2,6]. The taxon Ovalentaria is well supported by molecular data, although the relationships of major ovalentarian taxa remain unresolved and support values for many basal nodes are very low [1,2,6,54].

For the first time, the phylogenetic relationships of the Ovalentaria are analysed using morphological data. This study aims to (i) compare the caudal-fin skeleton of ovalentarian taxa based on detailed descriptions, (ii) reconstruct the evolution of the caudal-fin skeleton within the Ovalentaria using the phylogenetic hypothesis of Betancur-R *et al*. [2], (iii) discuss functional aspects related to locomotion modes and (iv) construct a phylogenetic hypothesis fitting the evolution of the examined morphological data. The agreements and discrepancies between the molecular and morphological topologies are discussed in light of the caudal-fin evolution.

## 2. Material and methods

### 2.1. Taxonomic sampling and morphological analysis

The caudal-fin skeleton of 355 species were examined and/or reviewed from literature in this study (electronic supplementary material, S1). We studied the caudal-fin skeleton of 275 species from a total of 556 cleared and stained specimens, X-ray images or µCT scans from the collections of the Australian Museum (AMS), the Deutsches Meeresmuseum (DMM), the Florida Museum of Natural History (FLMNH), the Muséum national d'Histoire naturelle (MNHN), the Phyletisches Museum Jena (PMJ) and the Zoologische Staatssammlung München (ZSM). Furthermore, data on the caudal-fin skeletons of 203 species were collected by reviewing literature (electronic supplementary material, S1) [33,45,46,50,56–97]. The taxon sample covers all ovalentarian families and, if possible, we selected taxa from different phylogenetic positions within each family.

During this study, specimens were cleared and double stained (bone stained in red and cartilage in blue) following the protocols by Dingerkus & Uhler [98] and Taylor & Van Dyke [99]. Larval material examined in this study was cleared and stained following the protocol by Schnell *et al.* [100]. Pictures of the caudal-fin skeleton were taken either with a Canon EOS 80D and a Canon MP-E 65 mm macro lens, an Axiocam microscope camera attached to a Zeiss Discovery V20 stereomicroscope, or a Leica M205 stereoscope with a DMC 4500 camera. Pictures were processed in Adobe Photoshop and Zeiss ZEN software and plates were assembled in Adobe Illustrator.

Thirty-eight characters of the caudal-fin skeleton were evaluated for each species during this study (data matrix available in electronic supplementary material, S1). Definitions of each character and the respective states are available in electronic supplementary material, S2. Based on the character states identified for each species, we reconstructed a ground plan for each ovalentarian family. To do so, we used the latest phylogenetic hypotheses of the respective family if available [75,87,101–110], otherwise phylogenetic relationships presented in Betancur-R *et al*. [2] were used, in combination with the principle of parsimony. If this procedure brought forth an ambiguous result due to a low number of examined taxa, we considered less or unfused character states (in applicable characters) to be preferred over fused character states in the ground plan reconstruction. The reconstructed ground plan was then used in further analyses and is the basis for the descriptions of the caudal-fin skeletons of the ovalentarian families given herein. Species that differ in character states from the reconstructed ground plan of their respective family are discussed following each family description.

For all analyses performed in this study, we selected Polymixiidae, Berycimorphaceae and Holocentrimorphaceae as outgroups. These taxa are most closely related to the Percomorpha, in which the Ovalentaria are grouped. In previous studies, analysing the phylogeny of percomorphs using morphological data, these taxa were established as suitable outgroups [9]. Other possible outgroup taxa from within the Percomorpha, e.g. Pelagiaria or Eupercaria, were ineligible during this study because of their great variation of caudal-fin skeletons [40,46]. Finding a suitable representative with a caudal-fin skeleton similar to the ground plan of the respective taxon was not possible during this study.

### 2.2. Ancestral character state estimation

Ancestral character state estimation was performed in RStudio using the packages ape [111], Geiger [112] and phytools [113] and the phylogenetic tree provided by Betancur-R *et al*. [2] as basis for the analysis. First, the phylogenetic tree provided by Betancur-R *et al*. [2] was trimmed to only the Ovalentaria and the outgroup taxa. Then all ovalentarian taxa were further trimmed to family level except atheriniforms and cyprinodontiforms, which were reduced to their most-recent common ancestor, as the provided tree did not represent the full diversity of families of these taxa. Afterwards, the best-fitting parameters (i.e. model, pi-value and transformation matrix) for each character were determined using a customized script mainly employing the functions *fitMk* (phytools) and *fitDiscrete* (Geiger). Multistate characters were not considered ordered *a priori*. Ancestral character state estimation was performed using the *make.simmap* function (phytools) and a modified version of the *describe.simmap* function (phytools). For plotting trees, the packages ape [111] and ggtree [114] were used.

### 2.3. Phylogenetic analysis

The 48 ovalentarian families were examined, of which all atheriniform, beloniform and cyprinodontiform families were condensed as Atheriniformes, Beloniformes and Cyprinodontiformes, respectively, as their monophyly was confirmed by both morphological and molecular-genetic data (e.g. [2,7,102,104,115]). The compiled data were analysed with parsimony (MP), maximum likelihood (ML) and Bayesian inference (BI) approaches.

Parsimony analyses were conducted with TNT v. 1.5 [116,117]. Heuristic searches were carried out with Traditional search (Wagner trees: 500 replicates and TBR: 350 replications) and New Technology search algorithms (RAS: 5000 additive sequences, Sectorial Search: RSS + CSS (500 rounds) with minimal sector size 5, Ratchet: 500 iterations, Drift: 500 cycles and Tree fusing: 20 rounds). Node supports were calculated via bootstraping (1000 replications using Traditional search) with support values given as frequency differences [118].

ML analyses were performed in iqtree2 [119]. First, the ModelFinder algorithm [120] was used to select the optimal model for the subsequent phylogenetic estimation process. Second, the ML phylogenetic analysis was run including the ultrafast bootstrap approximation [121] with 1 000 000 bootstrap replicates to compute branch support values.

BI analyses were conducted in MrBayes 3.2.7a [122] employing the CIPRES Science Gateway [123]. The Mkv model (with rates of the character evolution model set to a lognormal distribution) with one partition was run under the following settings: four separated runs each with one cold and five heated chains, three swaps and heated chain temperature set to 0.09; burn-in fraction set at 0.25 for 10^7^ generations sampled every 1000 generations. The function ‘run BEAGLE’ in CIPRES was activated for the analyses [124]. The consensus topology was calculated under the majority rule together with the posterior probabilities of each node.

### 2.4. Terminology

The terminology of the skeletal elements of the caudal fin generally follows Schultze & Arratia [21] and Fujita [125]. Below, terms are briefly defined or, if differing from the above, explained in detail:

Compound centrum (CC): most posterior vertebra to which the lower and upper hypurals are connected (articulated or fused); the anterior portion resembles a half centrum and the posterior portion is cone-shaped and may bend upwards. The CC is not a phylogenetically defined term because development of this structure varies greatly (either one ural centrum (UC) develops or two ural centra develop that later fuse), but results in similar adult morphologies. Therefore, it is an anatomical term not implying homology between taxa.

Hypural diastema (HD): space between hypural 2 and hypural 3.

Epural (Ep): detached neural spine (NS) previously associated with neural arch (NA) of preural or UC. When several epurals are present, these are numbered from anterior to posterior. Numbers do not imply homology.

Haemal arch (HA): ventral attachment to caudal vertebra enclosing the arteria caudalis developing from paired basiventral cartilages.

Haemal spine (HS): spine-like, ventral extension of the fused tips of the left and right halves of the HA or cartilaginous preformed element that fuses to the tips of the HA during ontogeny.

Hypurapophysis (HU): attachment site for the hyperchordal longitudinalis muscles bilaterally on the parhypural (PH).

Hypural (Hyp): modified HS without HA that is associated with a UC or the CC (either articulated or fused).

Inter-haemal spine cartilage (IHC): cartilaginous element posterior to tip of the respective HS (indicated by respective number). In some cases, in close proximity to the following HS.

Inter-neural spine cartilage (INC): cartilaginous element posterior to tip of the respective NS (indicated by respective number). In some cases, in close proximity to the following NS.

Lower hypural plate (LHP): hypural element ventral to the diastema originating either by fusion of cartilaginous precursors of hypural 1 and hypural 2 or from one single cartilaginous precursor.

Neural arch (NA): dorsal attachment to vertebra enclosing the spinal cord developing from paired basidorsal cartilages.

Neural spine (NS): spine-like, dorsal extension of the fused tips of the left and right halves of the NA or cartilaginous preformed element which fuses to the tips of the NA during ontogeny. A reoccurring character state of the NS of preural centrum 2 is that it is shortened. The normal length of this NS is defined as at least as long as the NS of first non-preural NS; shortening can occur in two states: short (greater than 50% normal length) or truncated (less than 50% normal length).

Parhypural (PH): HA and HS or only HS anterior to Hyp1. The HA of the PH, if present, provides the exit point of the arteria caudalis.

Preural centrum (PU): vertebral centrum anterior to the ural centra/CC that supports caudal-fin rays with its haemal and/or NSs. Preural centra are counted from posterior to anterior. Preural centrum 1 is the most posterior PU and, if present, supports the PH.

Upper hypural plate (UHP): hypural element dorsal to the diastema originating either from fusion of cartilaginous precursors of hypural 3 and hypural 4 or from one single cartilaginous precursor. Hypural 5 can additionally be included into the UHP.

Ural centrum (UC): centrum at the posterior end of the vertebral column characterized by absence of HAs and supporting hypurals ventrally.

Uroneural (UN): paired, elongated bones dorsal to the ural centra/CC and dorso-lateral to the notochord; evolutionarily derived from ural NAs.

## 3. Results

### 3.1. Morphology of the caudal-fin skeleton

Below we present the composition of the caudal-fin skeleton of ovalentarian families. A summary of the ground plan is provided and variations are reported.

### 3.1.1. Atheriniformes

**Figure 2.**
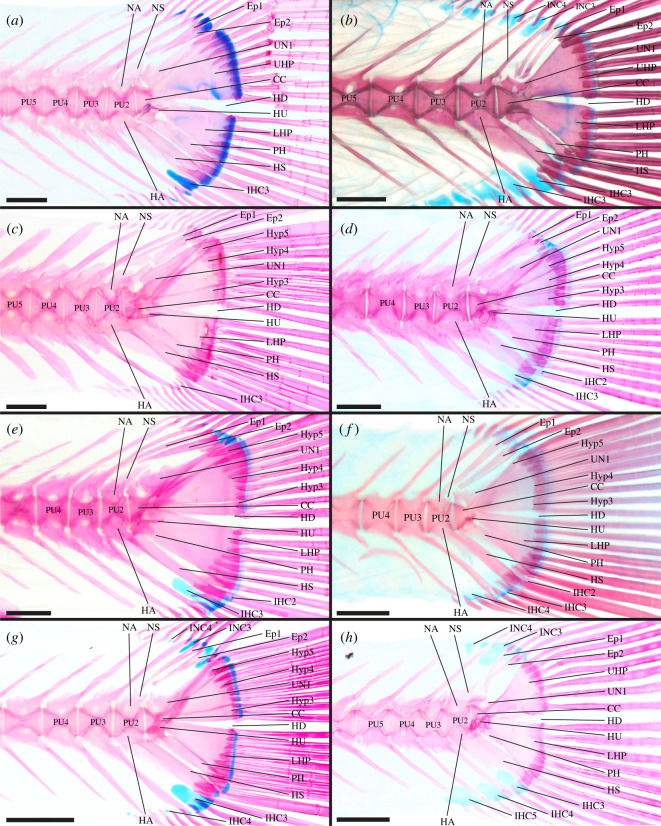
Caudal-fin skeleton of cleared and stained specimens of atheriniform families: (*a*) Atherinopsidae—*Menidia conchorum* (DMM IE/11399, SL = 65.5 mm); (*b*) Phallostethidae—*Gulaphallus mirabilis* (MNHN 2020 0379, SL = 24.2 mm); (*c*) Atherinidae—*Atherina boyeri* (DMM IE/16473, SL = 63.9 mm); (*d*) Bedotiidae—*Bedotia geayi* (DMM IE/15880, SL = 78.1 mm); (*e*) Melanotaeniidae—*Glossolepis incisa* (DMM IE/12202, SL = 45.7 mm); (*f*) Melanotaeniidae—*Iriatherina werneri* (DMM IE/11407, SL = 30.4 mm); (*g*) Telmatherinidae—*Marosatherina ladigesi* (DMM IE/11011, SL = 35.5 mm); (*h*) Pseudomugilidae—*Pseudomugil furcatus* (DMM IE16311, SL = 29.4 mm). CC, compound centrum; HD, hypural diastema; Ep, epural; IHC, inter-haemal spine cartilage; INC, inter-neural spine cartilage; HA, haemal arch; HS, haemal spine; HU, hypurapophysis; Hyp, hypural; LHP, lower hypural plate; NA, neural arch; NS, neural spine; PH, parhypural; PU, preural centrum; UHP, upper hypural plate; UN, uroneural. Scale bar = 1 mm.

**Atherinopsidae**—6 out of 13 genera examined [37,46,50], e.g. *Menidia conchorum* (figure 2*a*).

CC, PU2, PU3, PU4 and PU5 contribute to the caudal fin; CC contains UC1 + UC2 [CC (UC1 + UC2)]; Hyp1 + Hyp2 fused to form LHP, LHP fused to CC; Hyp3 and Hyp4 separate, Hyp3 and Hyp4 articulate with CC; Hyp5 present; Hyp5 not fused to Hyp4 nor to CC; PH articulates with CC, not fused to LHP; HU present on PH, HU splint-like and directed posteriorly; UN present, UN fused to Hyp5; two Ep present; HA of PU2 and HA of PU3 fused to respective centrum; NS of PU2 truncated; IHC3 and IHC4 present, INC3 and INC4 present.

*Leuresthes tenuis* has two ural centra (U1 + U2) in early ontogeny that later fuse to form the CC [68,70]. In *Atherinella eriarcha* the PH is fused to the LHP. Hyp3 + Hyp4 are fused to form a UHP in *A. eriarcha*, *Menidia beryllina* and *M. conchorum* (figure 2*a*). Additionally, the UHP is fused to the CC in *A. eriarcha* and *M. beryllina*. In *A. brasiliensis*, *A. eriarcha*, *M. beryllina* and *M. conchorum* Hyp5 is fused to the UHP as well as the CC. The HD is anteriorly restricted in *A. brasiliensis* and almost absent in in *Atherinops affinis* due to the close connection of the LHP and Hyp3 over almost their whole length. UN is fused to both the CC and Hyp5 in *Menidia* while in *Odontesthes bonariensis* both fusion to only the CC and unfused UN were found. IHC2 and IHC5 are present in *O. bonariensis*. In *Membras martinica* only IHC4 and in *M. conchorum* only IHC3 is present.

**Atherionidae**—1 out of 1 genus examined [46].

CC, PU2, PU3, PU4 and PU5 contribute to the caudal fin; components of CC unknown [CC (?)]; LHP present, LHP fused to CC; Hyp3 + Hyp4 fused to form UHP, UHP articulates with CC; Hyp5 present, Hyp5 fused to CC; PH articulates with CC, not fused to LHP; HU present on PH, HU splint-like, short (not reaching the posterior border of the PH) and pointing posteriorly; UN present, UN fused to Hyp5; two Ep present; HA of PU2 and PU3 fused to respective centrum; NS of PU2 truncated; IHC3 and IHC4 present.

*Atherion elymus* has two Ep while in *A. maccullochi* only one Ep is present.

**Phallostethidae**—4 out of 4 genera examined [58,62], e.g. *Gulaphallus mirabilis* (figure 2*b*).

CC, PU2, PU3, PU4 and PU5 contribute to the caudal fin; CC contains UC1 + UC2 [CC (UC1 + UC2)]; LHP present, LHP fused to CC; Hyp3 + Hyp4 fused to form UHP, UHP fused to CC; Hyp5 either fused into UHP or absent; PH articulates with CC, not fused to LHP; UN present, UN fused to CC and UHP; two Ep present; HA of PU2 and HA of PU3 fused to respective centrum; NS of PU2 normal length; IHC3, IHC4, IHC5 and IHC6 present, INC2, INC3 and INC4 present.

In *Neostethus lankesteri* and *Phenacostethus smithi* PU6 also contributes to the caudal fin. Parenti [62] did not report a NS on PU2 for *Phallostethus dunckeri*. In *Gulaphallus mirabilis* INC5 and INC6 are present (figure 2*b*).

**Isonidae**—1 out of 1 genus examined [46].

CC, PU2, PU3, PU4 and PU5 contribute to the caudal fin; components of the CC unknown [CC (?)]; Hyp1 + Hyp2 fused to form LHP, LHP fused to CC; Hyp3 + Hyp4 fused to form UHP, UHP articulates with CC; Hyp5 present, Hyp5 fused to UHP; PH fused to CC and to LHP; HU present on PH, HU splint-like and directed posteriorly; UN present, UN fused to Hyp5; one Ep present; HA of PU2 and HA of PU3 fused to respective centrum; NS of PU2 truncated; IHC3 present, INC absent.

In *Iso nesiotes* PU5 does not contribute to the caudal fin. Also, the NS of PU2 is not truncated.

**Atherinidae**—9 out of 13 genera examined [33,46,56,57], e.g. *Atherina boyeri* (figure 2*c*).

CC, PU2, PU3, PU4 and PU5 contribute to the caudal fin; CC contains UC1 + UC2 [CC (UC1 + UC2)]; Hyp1 + Hyp2 fused to form LHP, LHP fused to CC; Hyp3 and Hyp4 separate, Hyp3 and Hyp4 articulate with CC; Hyp5 present, Hyp5 not fused to CC nor to Hyp4; PH articulates with CC, not fused to LHP; HU present on PH, HU splint-like and directed postero-dorsally; UN present, UN not fused to CC or upper hypurals; 2 Ep present; HA of PU2 and HA of PU3 fused to respective centrum; NS of PU2 truncated; IHC3 present, INC4 present.

In many atherinid taxa Hyp3 and Hyp4 are fused to form the UHP (i.e. *Atherina harringtonensis*, *Atherinomorus vaigiensis*, *Craterocephalus amniculus*, *Doboatherina bleekeri*, *Hypoatherina barnesi*). Hyp5 of *C. amniculus*, *D. bleekeri* and *Teramulus kieneri* is fused to the CC. The HU of *C. amniculus* and *C. eyresii* is shorter than in other atherinid species. In *Atherina breviceps*, *Atherinomorus stipes*, *C. honoriae* and *Leptatherina wallacei* the UN is fused to the CC. The UN of *D. bleekeri*, *H. barnesi*, *Kestratherina esox*, *L. presbyteroides* and *T. kieneri* is additionally fused to Hyp5. IHC4 can additionally be present, i.e. *Atherinomorus* and *Doboatherina*, while INC3 is missing in *Atherina*, *C. honoriae*, *L. wallacei* and *T. kieneri*.

**Bedotiidae**—2 out of 2 genera examined [57,65–67,70], e.g. *Bedotia geayi* (figure 2*d*).

CC, PU2, PU3 and PU4 contribute to the caudal fin; CC contains UC1 + UC2 [CC (UC1 + UC2)]; Hyp1 + Hyp2 fused to form LHP, LHP fused to CC; Hyp3 and Hyp4 separate, Hyp3 and Hyp4 articulate with CC; Hyp5 present, Hyp5 articulates with CC, Hyp5 not fused to Hyp4; PH articulates with CC, not fused to LHP; HU present on PH, HU splint-like and directed posteriorly; UN present, UN fused to CC; two Ep present; HA of PU2 and PU3 fused to respective centrum; NS of PU2 truncated; IHC3 present, INC absent.

In *Rheocles alaotrensis* and *R. derhami* Hyp4 and Hyp5 are fused. In the genus *Rheocles* (except *R. vatosoa*) the PH is partially or completely fused to the LHP. In *R. sikorae* and *R. vatosoa* the HD is anteriorly restricted. According to Stiassny [57] the UN is fused to both the CC and Hyp5; however, we were not able to observe such a fusion. IHC3 is absent in *R. pellegrini* and INC4 is absent in some *Bedotia geayi* (figure 2*d*). Presence and absence of IHC4 within Bedotiidae is erratically distributed, which did not allow the reconstruction of the character state in the most-recent common ancestor.

**Melanotaeniidae**—7 out of 7 genera examined [59,60,70,126], e.g. *Glossolepis incisa* (figure 2*e*) and *Iriatherina werneri* (figure 2*f*).

CC, PU2, PU3 and PU4 contribute to the caudal fin; CC contains UC1 + UC2 [CC (UC1 + UC2)]; Hyp1 + Hyp2 fused to form LHP, LHP fused to CC; Hyp3 and Hyp4 separate, connection of Hyp3 and Hyp4 to CC inconclusive; Hyp5 present, connection of Hyp5 to CC inconclusive, Hyp5 fused to Hyp4; connection of PH and CC inconclusive, PH fused to LHP; HU present on PH, HU splint-like and directed posteriorly; UN present, UN fused to CC; two Ep present; HA of PU2 and PU3 fused to respective centrum; NS of PU2 truncated; IHC3 + IHC4 present, INC absent.

In *Chilatherina axelrodi* PU5 also contributes to the caudal fin. Hyp3 and Hyp4 are fused in *Rhadinocentrus ornatus* and *Pelangia mbutaensis* forming the UHP. In *C. axelrodi* and *Glossolepis incisa* (figure 2*e*) Hyp5 is not fused to Hyp4. The type of connection of the PH and Hyp3 to Hyp5 with the CC remains inconclusive because in *Iriatherina werneri* (figure 2*f*) we observed a fused condition while in later branching taxa these elements articulate, and we were not able to retrieve an unambiguous character state for the earliest branching melanotaeniid *R. ornatus*. The HD in *Melanotaenia nigrans* is anteriorly restricted. The UN remains unfused in *C. axelrodi*. In *M. nigrans* only one Ep is present. IHC2 is present in *G. incisa* (figure 2*e*) and *I. werneri* (figure 2*f*). IHC4 is absent in *G. incisa* and *R. ornatus*. Based on the latest molecular hypothesis, the species *Cairnsichthys rhombosomoides* is no longer considered a melanotaeniid and is closer related to telmatherinids and pseudomugilids [102]. It differs from melanotaeniids in having the PH separated from the LHP.

**Telmatherinidae**—2 out of 5 genera examined [63], e.g. *Marosatherina ladigesi* (figure 2*g*).

CC, PU2, PU3 and PU4 contribute to the caudal fin; CC contains UC1 + UC2 [CC (UC1 + UC2)]; Hyp1 + Hyp2 fused to form LHP, LHP fused to CC; Hyp3 and Hyp4 separate, Hyp3 and Hyp4 articulate with CC; Hyp5 present, Hyp5 articulates with CC, Hyp5 not fused to Hyp4; PH articulates with CC, PH not fused to LHP; HU present on PH, HU splint-like and directed posteriorly; UN present, UN fused to CC; two Ep present; HA of PU2 and PU3 fused to respective centrum; NS of PU2 truncated; IHC3, IHC4 and IHC5 present, INC3 and INC4 present.

In half of the examined specimens of *Marosatherina ladigesi* Hyp4 and Hyp5 are fused. No IHC or INC are reported for *Kalyptatherina helodes*.

**Pseudomugilidae**—3 out of 3 genera examined [64,70], e.g. *Pseudomugil furcatus* (figure 2*h*).

CC, PU2, PU3 and PU4 contribute to the caudal fin; CC contains UC1 + UC2 [CC (UC1 + UC2)]; LHP present, LHP fused to CC; UHP present, UHP fused to CC; Hyp5 absent; PH articulates with CC, PH part of the LHP; HU present on PH, HU spur-like and directed laterally; UN present, UN fused to UHP and CC; two Ep present; HA of PU2 and PU3 fused to respective centrum; NS of PU2 truncated; IHC3, IHC4 and IHC5 present, INC3 and INC4 present.

In *Pseudomugil furcatus* the PH develops as part of the LHP. In *P. majusculus* and some specimens of *P. signifer* the PH is separated from the LHP, which indicates the individual development of the PH. Therefore, it is possible that in the ground plan of pseudomugilids the PH still develops as an individual element.

**Dentatherinidae**—1 out of 1 genus examined [127].

CC, PU2, PU3 and PU4 contribute to the caudal fin; CC probably contains UC1 + UC2 [CC (UC1 + UC2)]; Hyp1 + Hyp2 fused to form LHP, LHP fused to CC; Hyp3 + Hyp4 fused to form UHP, UHP articulates with CC; Hyp5 present, Hyp5 articulates with CC, Hyp5 not fused to UHP; PH articulates with CC, PH not fused to LHP; HU present on PH, HU directed antero-ventrally; UN present, UN fused to Hyp5; one Ep present; HA of PU2 and PU3 fused to respective centrum; NS of PU2 truncated, IHC and INC absent.

### 3.1.2. Beloniformes

**Figure 3.**
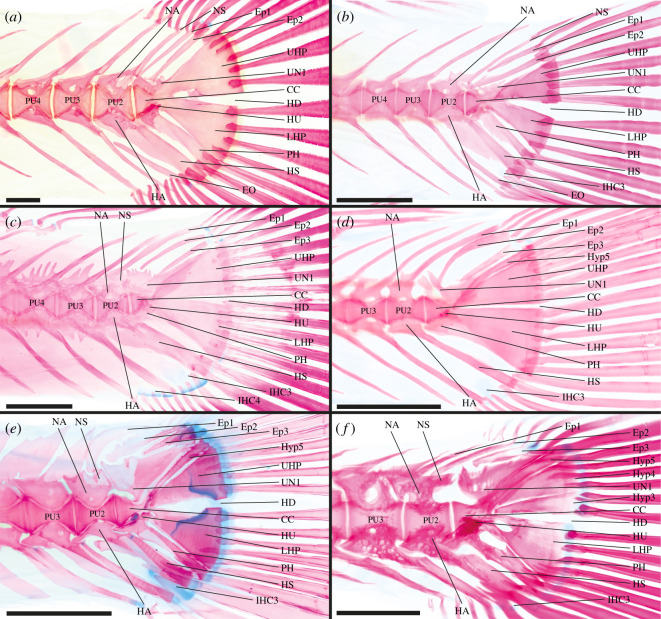
Caudal-fin skeleton of cleared and stained specimens of beloniform families: (*a*) Adrianichthyidae—*Adrianichthys oophorus* (ZFMK unregistered, SL = 58.0 mm), (*b*) Adrianichthyidae—*Oryzias sinensis* (DMM IE/16499, SL = 22.6 mm); (*c*) Zenarchopteridae—*Hemirhamphodon phaiosoma* (DMM IE/16679, SL = 51.8 mm); (*d*) Zenarchopteridae—*Dermogenys siamensis* (DMM IE/16502, SL = 27.2 mm); (*e*) Hemiramphidae—*Hyporhamphus limbatus* (DMM IE/16677, SL = 32.5 mm); (*f*) Belonidae—*Belone belone* (DMM IE/16519, SL = 84.7 mm). CC, compound centrum; HD, hypural diastema; Ep, epural; EO, extra caudal ossicle; IHC, inter-haemal spine cartilage; HA, haemal arch; HS, haemal spine; HU, hypurapophysis; Hyp, hypural; LHP, lower hypural plate; NA, neural arch; NS, neural spine; PH, parhypural; PU, preural centrum; UHP, upper hypural plate; UN, uroneural. Scale bar = 1 mm.

**Adrianichthyidae**—2 out of 2 genera examined [46,50,56,61,70], e.g. *Adrianichthys oophorus* (figure 3*a*) and *Oryzias sinensis* (figure 3*b*).

CC, PU2, PU3 and PU4 contribute to the caudal fin; CC contains UC1 + UC2 [CC (UC1 + UC2)]; LHP present, LHP fused to CC; UHP present, UHP fused to CC; Hyp5 absent; PH articulates with CC, PH not fused to LHP; HU present on PH, HU stout dorsal projection; UN present, UN fused to CC; two Ep present; HA of PU2 and PU3 fused to respective centrum; NS of PU2 normal length; IHC3 present, INC absent; extra caudal ossicle (EO) present between HS of PU2 and PH.

In *Oryzias sinensis* and *O. woworae* PU4 does not contribute to the caudal fin (figure 3*b*). In *O. sarasinorum* only one Ep is present.

**Zenarchopteridae**—5 out of 5 genera examined [50,73], e.g. *Hemirhamphodon phaiosoma* (figure 3*c*) and *Dermogenys siamensis* (figure 3*d*).

CC, PU2 and PU3 contribute to the caudal fin; components of CC unknown [CC (?)]; Hyp1 + Hyp2 fused to form LHP, LHP fused to CC; Hyp3 + Hyp4 fused to form UHP, UHP fused to CC; Hyp5 present, Hyp5 fused to Hyp4/UHP and CC; connection of PH and CC inconclusive, PH fused to LHP; UN present, UN fused to CC; three Ep present; HA of PU2 and PU3 fused to respective centrum; NS of PU2 truncated; IHC3 present, INC absent.

In *Hemirhamphodon phaiosoma* PU4 additionally contributes to the caudal fin (figure 3*c*). In *Dermogenys pusilla*, *D. siamensis* (figure 3*d*) and *H. phaiosoma* the UHP articulates with the CC. In many zenarchopterid species, the LHP and UHP are grown towards each other, resulting in a narrowed HD. In *H. kuekenthali* the LHP and UHP are even partially fused. In *Nomorhamphus* (except *N. kolonodalensis* and *N. viviparus*) Hyp5 is no longer distinguishable from the UHP. Based on the fusion of Hyp5 to the UHP and the CC in other zenarchopterids, it is highly plausible that Hyp5 is fused to the UHP in these species. In *Dermogenys*, *N. kolonodalensis* and *N. viviparus* Hyp5 is not fused to the UHP and in *D. pusilla* and *D. siamensis* (figure 3*d*) it articulates with the CC. Meisner [73] reported that in the zenarchopterids (*H. kuekenthali*, *N. viviparus*, *Tondanichthys kottelati* and *Zenarchopterus rasori*) the PH articulates with the CC. However, in the herein examined zenarchopterids (except *Dermogenys*), the PH is fused to the CC. The PH is not fused to the LHP in *T. kottelati* and *Z. rasori*. UN is fused only to the UHP in *T. kottelati* and *Z. rasori* and fused to the CC and the UHP in *Nomorhamphus* and *H. kuekenthali*. IHC3 is absent in *Z. rasori* and IHC4 is present in *D. pusilla*, *N. lanceolatus*, *N. liemi* and *N. megarrhamphus*.

**Hemiramphidae**—3 out of 8 genera examined [46,70,72–74], e.g. *Hyporhamphus limbatus* (figure 3*e*).

CC, PU2, PU3 and PU4 contribute to the caudal fin; CC contains UC1 + UC2 [CC (UC1 + UC2)]; Hyp1 + Hyp2 fused to form LHP, LHP fused to CC; Hyp3 + Hyp4 fused to form UHP, UHP articulates with CC; LHP and UHP grown towards each other posteriorly; Hyp5 present, Hyp5 articulates with CC, Hyp5 not fused to UHP; PH articulates with CC, PH not fused to LHP; HU present on PH, HU broad lateral projection; UN present, UN fused to CC, UN expanded antero-dorsally; three Ep present; HA of PU2 and PU3 fused to centrum; NS of PU2 truncated; IHC and INC absent.

Based on molecular analyses, the Hemiramphidae do not form a monophyletic taxon [2,103]. The genera *Euleptorhamphus*, *Hemiramphus* and *Oxyporhamphus* are more closely related to the Exocoetidae, while the genera *Arrhamphus* and *Hyporhamphus* are more closely related either to the Zenarchopteridae and Belonidae [103] or to the taxon formed by Exocoetidae and the remaining Hemiramphidae [2]. The caudal-fin skeletons of both subgroups do not vary and are described by the above-mentioned characters. The only difference is the presence of IHC3 in the ground plan of the last common ancestor of *Arrhamphus* and *Hyporhamphus*.

The elements of the caudal skeleton are in general expanded in the medial axis, which is particularly visible in the enlarged NA, NS, HA and HS (figure 3*e*). In an examined specimen of *Hyporhamphus limbatus* Hyp5 is fused to the UHP (figure 3*e*). Rosen [50] depicted that Hyp5 is fused to the CC in *Chriodorus atherinoides*. In *Hemiramphus brasiliensis* the PH is fused to the CC. Due to this fusion the HU is positioned lateral to the CC; however, we still regard it to be part of the PH and to be one result of this fusion. Fujita [46] reported that in *Hy. sajori* the HA of PU2 articulates with the centrum, but the drawings of Lee *et al.* [72] suggest that these elements are fused, which would correspond to all other examined hemiramphids. IHC2 is present in *Hy. sajori* and can be present in *Hy. limbatus*. In *Hy. picarti* IHC3 is absent, and in *Hy. limbatus* IHC4 is present.

**Exocoetidae**—3 out of 7 genera examined [46,50,71,128].

CC, PU2, PU3, PU4, PU5 (and PU6) contribute to the caudal fin; CC probably contains one UC [CC (UC1/UC2?)]; LHP present, LHP articulates with CC; UHP present, UHP articulates with CC; Hyp5 absent; PH fused to CC, PH not fused to LHP; HU present lateral on CC due to fusion of PH and CC, HU directed laterally and posteriorly; UN present, UN fused to CC and extremely enlarged; three Ep present; HA of PU2 and PU3 fused to respective centrum; NS of PU2 short; IHC and INC absent.

The elements of the caudal skeleton are in general expanded which is particularly visible in the enlarged NA, NS, HA and HS. Ontogenetic data from *Cheilopogon doederleinii* suggest that only one UC develops [71]. It cannot be determined if this UC represents UC1, UC2 or if it is a product of evolutionary fusion of these two UC. In small specimens of *Parexocoetus brachypterus* and *C. doederleinii* the PH still articulates with the CC. In *P. brachypterus* the NS of PU2 is truncated. IHC2 is present in *C. doederleinii*.

**Belonidae**—6 out of 12 genera examined [46,69], e.g. *Belone belone* (figure 3*f*).

CC, PU2, PU3 and PU4 contribute to the caudal fin; CC probably contains UC1 + UC2 [CC (UC1 + UC2)]; Hyp1 + Hyp2 fused to form LHP, LHP fused to CC; Hyp3 + Hyp4 fused to form UHP, UHP articulates with CC; Hyp5 present, Hyp5 not fused to UHP or CC; PH articulates with CC, PH not fused to LHP; HU present on PH, HU stout lateral projection; UN present, UN fused to CC, UN antero-dorsally expanded; three Ep present; HA of PU2 and PU3 fused to respective centrum; NS of PU2 truncated; IHC absent, INC absent.

The elements of the caudal skeleton are in general expanded which is particularly visible in the enlarged NA, NS, HA and HS (figure 3*f*). In *Strongylura anastomella* and *Potamorrhaphis guianensis* PU2 and PU3 support the caudal fin. In *Tylosurus crocodilus* and many *Belone belone* specimens Hyp3 and Hyp4 are separate (figure 3*f*). The PH is fused to the LHP in *Cololabis saira*. In some *B. belone* specimens and in *Potamorrhaphis guianensis* there are only two Ep present. IHC3 is present in *B. belone* and *S. anastomella* and IHC4 is present in *B. belone*.

### 3.1.3. Cyprinodontiformes

**Figure 4.**
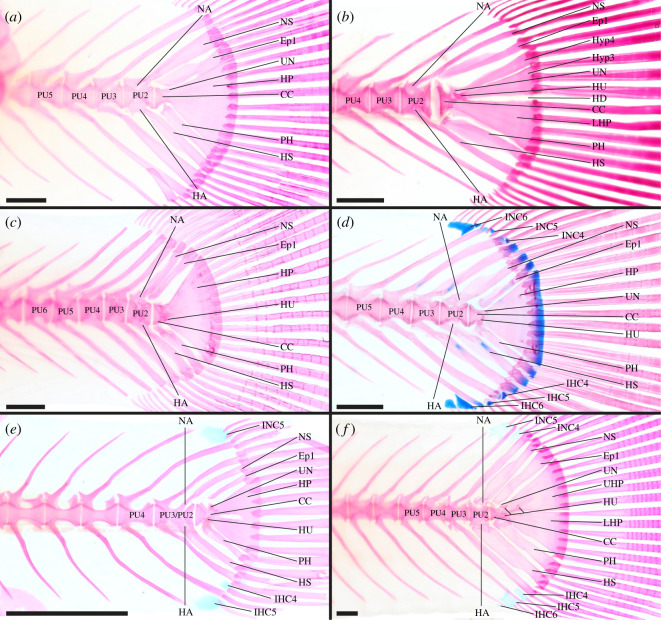
Caudal-fin skeleton of cleared and stained specimens of cyprinodontiform families: (*a*) Aplocheilidae—*Pachypanchax omalonotus* (DMM IE/16474, SL = 55.5 mm); (*b*) Nothobranchiidae—*Epiplatys togolensis* (DMM IE/11524, SL = 36.9 mm); (*c*) Goodeidae—*Ameca splendens* (DMM IE/16535, SL = 38.5 mm); (*d*) Fundulidae—*Fundulus cf. similis* (DMM IE/11142, SL = 41.7 mm); (*e*) Procatopodidae—*Aplocheilichthys spilauchen* (DMM IE/16539, SL = 14.1 mm); (*f*) Poecilidae—*Poecilia mexicana* (DMM IE/12190, SL = 52.4 mm). CC, compound centrum; HD, hypural diastema; Ep, epural; IHC, inter-haemal spine cartilage; INC, inter-neural spine cartilage; HA, haemal arch; HP, hypural plate; HS, haemal spine; HU, hypurapophysis; Hyp, hypural; LHP, lower hypural plate; NA, neural arch; NS, neural spine; PH, parhypural; PU, preural centrum; UHP, upper hypural plate; UN, uroneural. Scale bar = 1 mm.

**Aplocheilidae**—2 out of 2 genera examined [56,70], e.g. *Aplocheilus lineatus* (figure 4*a*).

CC, PU2, PU3, PU4 and PU5 contribute to the caudal fin; CC probably contains one UC [CC (UC1/UC2?)]; LHP present; Hyp3 + Hyp4 fused to form UHP; LHP + UHP fused to form large HP, HP fused to CC, HD absent; Hyp5 absent; PH articulates with CC, PH not fused to LHP; HU present on PH, HU triangularly extended dorsally; UN present, UN fused to CC or CC and HP; one Ep present; HA of PU2 and PU3 fused to respective centrum; NS of PU2 normal length; IHC and INC absent.

Ontogenetic stages of *Aplocheilus lineatus* suggest that one elongated UC develops anterior to the hypural plates [70]. It cannot be determined if this UC represents UC1, UC2 or if it is a product of evolutionary fusion of these two UC. In some juvenile specimens of *Aplocheilus lineatus* LHP, Hyp3 and Hyp4 are separate and not fused.

**Nothobranchiidae**—6 out of 14 genera examined [56,79], e.g. *Epiplatys togolensis* (figure 4*b*).

CC, PU2, PU3 and PU4 contribute to the caudal fin; components of CC unknown [CC (?)]; LHP present, LHP fused to CC; Hyp3 + Hyp4 fused to form UHP, UHP fused to CC; LHP and UHP close together, HD narrowed; Hyp5 absent; PH articulates with CC, PH not fused to LHP; HU absent; UN present, UN fused to CC and UHP; one Ep present; HA of PU2 and PU3 fused to respective centrum; NS of PU2 normal length; IHC and INC absent.

Hyp3 and Hyp4 are unfused in the few of the examined nothobranchiid species to form the UHP, i.e. *Epiplatys bifasciatus*, *E. togolensis* (figure 4*b*) and *E. sexfasciatus*. In some species the LHP and UHP are fused resulting in a large HP and a completely reduced HD (i.e. *Foerschichthys*, *Fundulopanchax* and *Nothobranchius*) while in *Pronothobranchius* only a partial fusion is observed. The PH is separate from the CC in *Aphyosemion bitaeniatum*, *E. annulatus*, *Foerschichthys*, *Nothobranchius* and *Pronothobranchius*.

**Rivulidae**—4 out of 39 genera examined [56,76,78,79].

CC, PU2, PU3 and PU4 contribute to the caudal fin; components of CC unknown [CC (?)]; LHP present, LHP fused to CC; Hyp3 + Hyp4 fused to form UHP, UHP fused to CC; Hyp5 absent; PH separate from CC, PH not fused to LHP; HU absent; UN present, UN fused to CC and UHP; one Ep present; HA of PU2 and PU3 fused to respective centrum; NS of PU2 normal length; IHC and INC absent.

In *Hypsolebias trilineatus* and *Spectrolebias semiocellatus* LHP and UHP are fused to form a large HP. As a result of this fusion the HD is absent. In *Anablepsoides bahianus* no UN is present.

**Profundulidae**—1 out of 2 genera examined.

CC, PU2, PU3 and PU4 contribute to the caudal fin; components of CC unknown [CC (?)]; LHP present, UHP present; LHP + UHP fused to form large HP, HP fused to CC, HD absent; Hyp5 absent; PH articulates with CC, PH not fused to LHP; HU present on PH; HU short and directed postero-dorsally; presence of UN uncertain; one Ep present; HA of PU2 and PU3 fused to respective centrum; NS of PU2 normal length; IHC and INC absent.

**Goodeidae**—1 out of 19 genera examined, e.g. *Ameca splendens* (figure 4*c*).

CC, PU2, PU3, PU4, PU5 and PU6 contribute to the caudal fin; components of CC unknown [CC (?)]; HP present, HP fused to CC; Hyp5 absent; PH articulates with CC, PH not fused to HP; HU present on CC; UN present, UN fused to CC and HP; one Ep present; HA of PU2 and PU3 fused to respective centrum; NS of PU2 normal length; IHC and INC absent.

**Fundulidae**—2 out of 3 genera examined [56,97], e.g. *Fundulus cf. similis* (figure 4*d*).

CC, PU2, PU3, PU4 and PU5 contribute to the caudal fin; components of CC unknown [CC (?)]; LHP present; UHP present; LHP and UHP fused to form large HP, HP fused to CC, HD absent; Hyp5 absent; PH articulates to CC, PH not fused to LHP; HU present on PH, HU directed dorsally (lateral to CC); UN present, UN fused to CC and HP; one Ep present; HA of PU2 and PU3 fused to respective centrum; NS of PU2 normal length; IHC4, IHC5 and IHC6 present, INC4, INC5 and INC6 present.

In the examined specimen of *Fundulus cf. jenkinsi* LHP and UHP are not completely fused, they are separate anteriorly and fused posteriorly. Further, at this early stage during ontogeny, the UN can be distinguished from the developing NA of the CC. In *Fundulus sciadicus* IHC3, and in *Lucania parva* IHC3 and INC3 are additionally present. In *Fundulus cf. jenkinsi* IHC4 and INC4 are absent.

**Valenciidae**—1 out of 1 genus examined [56].

CC, PU2, PU3 and PU4 contribute to the caudal fin; components of CC unknown [CC (?)]; LHP present; UHP present; LHP + HUP fused to form large HP, HP fused to CC; Hyp5 absent; PH articulates with CC, PH not fused to HP; HU present on PH; presence of UN uncertain; one Ep present; HA of PU2 and PU3 fused to respective centrum; NS of PU2 normal length; IHC4 present, INC3 and INC4 present.

In the specimen depicted in Costa [56, fig. 1c] an extra ossified structure is present in between the distal tips of HSPU2 and HSPU3.

**Cyprinodontidae**—2 out of 10 genera examined [56].

CC, PU2, PU3 and PU4 contribute to the caudal fin; components of CC unknown [CC (?)]; large HP present, HP fused to CC, HD absent; Hyp5 absent; PH separate from CC, PH not fused to HP; HU absent; UN present, UN fused to CC and HP; one Ep present; HA of PU2 and PU3 fused to respective centrum; NS of PU2 normal length; IHC3, IHC4 and IHC5 present, INC3, INC4 and INC5 present.

**Aphaniidae**—1 out of 8 genera examined [56].

CC, PU2, PU3 and PU4 contribute to the caudal fin; components of CC unknown [CC (?)]; LHP present; UHP present; LHP + UHP fused to form large HP, HP fused to CC, HD absent; Hyp5 absent; PH separate from CC, PH not fused to HP; presence of HU uncertain; UN present, UN fused to CC and HP; one Ep present; HA of PU2 and PU3 fused to respective centrum; NS of PU2 normal length; IHC4 and IHC5 present, INC5 present.

**Procatopodidae**—4 out of 14 genera examined [77], e.g. *Aplocheilichthys spilauchen* (figure 4*e*).

CC, PU2, PU3 and PU4 contribute to the caudal fin (figure 4*e*); CC contains one UC [CC (UC1/UC2?)]; LHP present; UHP present; LHP + UHP fused to form large HP, HP fused to CC, HD absent; Hyp5 absent; PH articulates with CC, PH not fused to HP; HU present on PH; UN present, UN fused to CC and HP, UN anteriorly with lateral projection; one Ep present; HA of PU2 and PU3 fused to respective CC; NS of PU2 normal length; IHC3, IHC4 and IHC5 present, INC4 and INC5 present.

It was shown that in *Poropanchax normani* one elongated UC develops anterior to the hypurals [70]. It cannot be determined if this UC represents UC1, UC2 or if it is a product of evolutionary fusion of these two UC. In *Lamprichthys tanganicanus* PU5 also contributes to the caudal fin. In *Micropanchax hutereaui* no HU was observable and INC3 is additionally present.

**Anablepidae**—1 out of 3 genera examined [46,56].

CC, PU2, PU3, PU4, PU5, PU6 and PU7 contribute to the caudal fin; components of CC unknown [CC (?)]; LHP present, LHP fused to CC; UHP present, UHP fused to CC; LHP + UHP partially fused, HD anteriorly restricted; Hyp5 absent; PH articulates with CC, PH not fused to LHP; HU present on PH, HU splint-like and directed postero-dorsally; UN present, UN fused to CC and UHP, UN anteriorly with lateral projection; one Ep present; HA of PU2 and PU3 fused to respective centrum; NS of PU2 normal length; IHC6, IHC7 and IHC8 present, INC6 and INC7 present.

In *Anableps dowii* only PU2 to PU6 contribute to the caudal fin. The HU in *A. dowii* and *A. anableps* are not anteriorly restricted.

**Poeciliidae**—7 out of 27 genera examined [46,56,77], e.g. *Poecilia mexicana* (figure 4*f*).

CC, PU2, PU3 and PU4 contribute to the caudal fin; CC probably contains one UC [CC (UC1/IC2?)]; LHP present; UHP present; LHP + UHP fused to form large HP, HP fused to CC, HD absent; Hyp5 absent; PH articulates with CC, PH not fused to HP; HU present on PH, HU triangular dorsal projection; UN present, UN fused to CC and HP, UN with lateral projection; one Ep present; HA of PU2 and PU3 fused to respective centrum; NS of PU2 normal length; IHC4 and IHC5 present, INC4 + INC5 present.

In *Poecilia mexicana* PU5 and in *Tomeurus gracilis* PU5 and PU6 additionally contribute to the caudal fin. In *Hiatirhaphis cascajalensis*, *Pamphorichthys hollandi*, *Po. mexicana* (figure 4*f*) and *Po. formosa* the LHP and the UHP are in close contact but are not fused. Costa [77] depicted the PH fused to CC and HP in *Fluviphylax zonatus* and *Pa. hollandi*. We were not able to observe this in any other poecilid. Rather, in *Po. formosa* and *Po. sphenops* the PH is separate from the CC. The UN is absent in *H. cascajalensis*. In *Neoheterandria elegans* and *Pa. hollandi* the HU is absent. IHC4 and INC4 are absent in *Tomeurus gracilis*. IHC3 is present in *Gambusia affinis*, *N. elegans*, *Po. bifurca* and *Po. picta*. And INC3 is present in *F. zonatus* and *N. elegans*. An additional IHC6 is present in *Po. mexicana* (figure 4*f*) and *Po. sphenops*, and INC6 is additionally present in *Po. sphenops*.

### 3.1.4. Cichliformes

**Figure 5.**
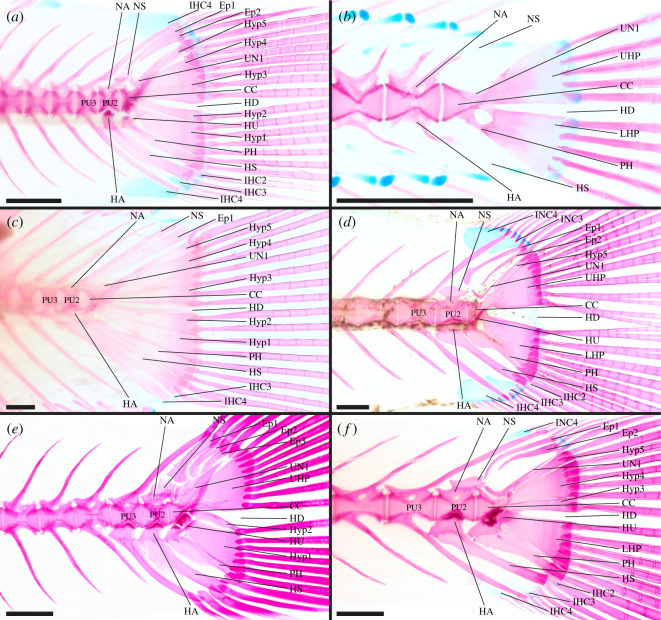
Caudal-fin skeleton of cleared and stained specimens of (*a*) Cichlidae—*Coptodon zillii* (DMM IE/16520, SL = 23.0 mm); (*b*) Pholidichthyidae—*Pholidichthys leucotaenia* (DMM IE/15795, SL = 57.5 mm); (*c*) Polycentridae—*Polycentrus schomburgkii* (DMM IE/13952, SL = 35.4 mm); (*d*) Ambassidae—*Ambassis dussumieri* (DMM IE/16526, SL = 36.2 mm); (*e*) Pomacentridae—*Chromis chromis* (DMM IE/11180, SL = 28.9 mm); (*f*) Mugilidae—*Myxus elongatus* (DMM IE/16697, SL = 38.0 mm). CC, compound centrum; HD, hypural diastema; Ep, epural; IHC, inter-haemal spine cartilage; INC, inter-neural spine cartilage; HA, haemal arch; HS, haemal spine; HU, hypurapophysis; Hyp, hypural; LHP, lower hypural plate; NA, neural arch; NS, neural spine; PH, parhypural; PU, preural centrum; UHP, upper hypural plate; UN, uroneural. Scale bar = 1 mm.

**Cichlidae**—19 out of 251 genera examined [45,46,70,93,129,130], e.g. *Coptodon zillii* (figure 5*a*).

CC, PU2 and PU3 contribute to the caudal fin; CC presumably contains one UC [CC (UC1/UC2?)]; Hyp1 and Hyp2 separated, Hyp1 and Hyp2 articulate with CC; Hyp3 and Hyp4 separated, Hyp3 and Hyp4 articulate with CC; Hyp5 present, Hyp5 articulates with CC, Hyp5 not fused to Hyp4; PH articulates with CC, PH not fused to Hyp1; HU present on PH, HU splint-like and directed postero-dorsally; UN present, UN not fused to CC or Hyp; two Ep present; HA of PU2 articulates with centrum, HA of PU3 fused to centrum; NS of PU2 truncated; IHC2, IHC3 and IHC4 present, INC4 present.

Ontogenetic data of different species (e.g. *Astatotilapia burtoni*, *Hemichromis bimaculatus*) indicate the development of one elongated UC anterior to the hypurals [70,130]. It cannot be determined if this UC represents UC1, UC2 or if it is a product of evolutionary fusion of these two UC. The anterior margins of Hyp2 and Hyp3 are close together in *Astronotus ocellatus*, *Cichla ocellaris* and *Oreochromis niloticus* which results in an anteriorly restricted HD. In two of the examined species (*Crenicichla saxatilis* and *Pterophyllum scalare*) Hyp3 and Hyp4 are fused to form the UHP which is fused to the CC. The HU is elongated in *Tilapia sparrmanii*, shortened in *Amatitlania nigrofasciata*, *A. ocellatus*, *Geophagus brasiliensis* and *P. scalare* and reduced to a ridge in *Apistogramma steindachneri*, *Cichlasoma portalegrense*, *Crenicichla* and *Mesonauta guyanae*. Only one Ep is present in *Astronotus ocellatus*. In *P. scalare* and *Apistogramma steindachneri* the HA of PU2 is fused to the centrum. IHC2 is missing in *P. scalare*, *Amatitlania nigrofasciata* and *Steatocranus* sp., while INC3 is present in *Chromidotilapia guntheri*, *Krobia guianensis* and *Mesonauta guyanae*, and INC4 is absent in *Guianacara owroewefi*.

### 3.1.5. Incertae sedis

**Pholidichthyidae**—1 out of 1 genus examined, e.g. *Pholidichthys leucotaenia* (figure 5*b*).

Only CC contributes to the caudal fin; components of CC unknown [CC (?)]; LHP present, LHP fused to CC; UHP present, UHP fused to CC; presence of Hyp5 questionable (either part of UHP or absent); PH fused to CC, PH fused to LHP; HU present on the PH, HU directed laterally, HU very short; UN absent; Ep absent; HA of PU2 and PU3 fused to respective centrum; HS of PU3 not connected to HA; NS of PU2 normal length, IHC and INC absent.

**Polycentridae**—4 out of 4 genera examined [75], e.g. *Polycentrus schomburgkii* (figure 5*c*).

CC, PU2 and PU3 contribute to the caudal fin; CC presumably forms from one UC [CC (UC1/UC2?)]; Hyp1 and Hyp2 separate, Hyp1 and Hyp2 articulate with CC; Hyp3 and Hyp4 separate, Hyp3 and Hyp4 fused to CC; Hyp5 present, Hyp5 not fused to Hyp4 nor to CC, Hyp5 shortened; PH articulates with CC, PH not fused to Hyp1; HU present on PH, HU directed laterally, HU short; UN present, UN not fused to CC nor to Hyp; one Ep present; HA of PU2 articulates with centrum, HA of PU3 fused to centrum; NS of PU2 normal length; IHC3 and IHC4 present, INC3 and INC4 present.

Small specimen of *Polycentropsis abbreviata* suggests that only one UC is developed during ontogeny based on similarities to cichlids and pomacentrids, which in comparable ontogenetic stages exhibit very similar morphologies. In *Monocirrhus polyacanthus* Hyp5 is severely shortened and Hyp2 and Hyp3 are close together leaving only a narrowed and anteriorly restricted HD. Otherwise, different combinations of reduced IHC and INC are observable.

**Ambassidae**—3 out of 7 genera examined, e.g. *Ambassis dussumieri* (figure 5*d*).

CC, PU2 and PU3 contribute to the caudal fin; components of CC unknown [CC (?)]; LHP present, LHP articulates with CC; UHP present, UHP fused to CC; Hyp5 present, Hyp5 not fused to UHP nor to CC; PH articulates with CC, not fused to LHP; HU present on PH, HU splint-like directed postero-dorsally; UN present, UN not fused to CC nor to UHP; three Ep present; HA of PU2 articulates with centrum, HA of PU3 fused to centrum; NS of PU2 truncated, outgrowth of membrane bone dorsal to NA; IHC2, IHC3 and IHC4 present, INC3 and INC4 present.

The LHP is fused to the CC in *Parambassis siamensis* and *Gymnochanda ploegi*. Only two Ep are present in *Ambassis dussumieri* (figure 5*d*) and one of the examined specimens of *Parambassis siamensis*. The HA of PU2 is fused to the centrum in *Parambassis* and *Gymnochanda*.

**Pomacentridae**—10 out of 29 genera examined [46], e.g. *Chromis chromis* (figure 5*e*).

CC, PU2 and PU3 contribute to the caudal fin; CC presumably forms from one UC [CC (UC1/UC2?)]; Hyp1 and Hyp2 separate, Hyp1 and Hyp2 articulate with CC; Hyp3 and Hyp4 separate, Hyp3 and Hyp4 fused to CC; Hyp5 present, Hyp5 not fused to Hyp4 nor to CC, Hyp5 shortened; PH articulates with CC, not fused to Hyp1; HU present on PH, HU directed postero-dorsally, HU thin and elongated; UN present, UN fused to CC, UN enlarged; three Ep present; HA of PU2 and HA of PU3 articulate with respective centrum; NS of PU2 truncated, outgrowth of membrane bone dorsal to NA; IHC2, IHC3 and IHC4 present, INC4 present.

During the ontogeny of *Amphiprion ocellaris* one elongated UC develops anterior to the hypurals. It cannot be determined if this UC represents UC1, UC2 or if it is a product of evolutionary fusion of these two UC. In some species the PH and Hyp1 (i.e. *Am. frenatus*, *Am. ocellaris* and *Pomachromis richardsoni*), Hyp1 and Hyp2 (i.e. *Abudefduf sexfasciatus*, *Am. frenatus*, *Am. ocellaris* and *P. richardsoni*), Hyp3 and Hyp4 (i.e. *Ab. sexfasciatus*, *Am. frenatus*, *Am. ocellaris*, *Dascyllus aruanus* and *P. richardsoni*) or/and Hyp4 and Hyp5 (i.e. *Am. ocellaris*) are fused. Hyp2 and Hyp3 (or the LHP and UHP, if present) grow towards each other in some species, resulting in an anteriorly restricted (i.e. *Ab. bengalensis*, *Ab. sexfasciatus*, *Ab. sordidus*, *Chromis chrysurus*, *Chromis notata*, *Pomacentrus coelestis* and *Pomacentrus rhodonotus*) or narrowed HD (i.e. *Amphiprion*). In many species Hyp5 is further shortened (i.e. *Ab. vaigiensis*, *Ab. sordidus*, *Ab. sexfasciatus*, *Cheiloprion labiatus*, *Chromis chromis*, *Chrysiptera leucopoma*, *D. aruanus*, *Plectroglyphidodon leucozonus*, *Pomacentrus*, *Stegastes nigricans*). Two instead of three Ep are present in *Pl. leucozonus*. In some species INC3 is present (i.e. *Ab. bengalensis*, *Ab. sexfasciatus*, *Chromis chrysura*, *Chromis notata* and *P. richardsoni*).

**Embiotocidae**—2 out of 13 genera examined [46].

CC, PU2 and PU3 contribute to the caudal fin; components of the CC unknown [CC (?)]; Hyp1 and Hyp2 separate, Hyp1 and Hyp2 articulate with CC; Hyp3 and Hyp4 separated, Hyp3 and Hyp4 articulate with CC, Hyp2 and Hyp3 closely together, HD anteriorly restricted along half the length of Hyp2; Hyp5 present, Hyp5 shortened, Hyp5 not fused to Hyp4 nor to CC; PH articulates with CC, PH not fused to Hyp1; HU present on PH; UN present, UN enlarged; three Ep present; HA of PU2 articulates with centrum, HA of PU3 fused to centrum; NS of PU2 short; IHC2, IHC3 and IHC4 present, INC3 and INC4 present.

### 3.1.6. Mugiliformes

**Mugilidae**—14 out of 26 genera examined [46,92,93,131,132], e.g. *Myxus elongatus* (figure 5*f*).

CC, PU2 and PU3 contribute to the caudal fin; CC forms from U1 + U2 [CC (U1 + U2)]; Hyp1 + Hyp2 fused to form LHP, LHP articulates with CC; Hyp3 and Hyp4 separate, Hyp3 and Hyp4 fused to CC; Hyp5 present, Hyp5 not fused to Hyp4 nor to CC; PH articulates with CC, not fused to LHP; HU present on PH, HU splint-like and directed postero-dorsally; UN present, UN not fused to CC nor to Hyp5 or UHP; two Ep present; HA of PU2 articulates with centrum, HA of PU3 fused to centrum; NS of PU2 truncated, outgrowth of membrane bone dorsal to NA; IHC2, IHC3 and IHC4 present, INC4 present.

Hyp3 and Hyp4 are fused in *Chelon*, *Dajaus*, *Ellochelon*, *Mugil*, *Neomyxus*, *Oedalechilus*, *Paramugil*, *Planiliza*, *Plicomugil* and *Rhinomugil*. The NS of PU2 is only short in *Mugil curema* and *M. incilis*. In some *Paramugil* specimens IHC2 is missing. INC 3 is present in *Dajaus monticola* and *Paramugil georgii*. In *Dajaus monticola* INC4 is absent while in *Aldrichetta forsteri* and *Myxus elongatus* (figure 5*f*) only some specimens miss INC4.

### 3.1.7. Incertae sedis

**Figure 6.**
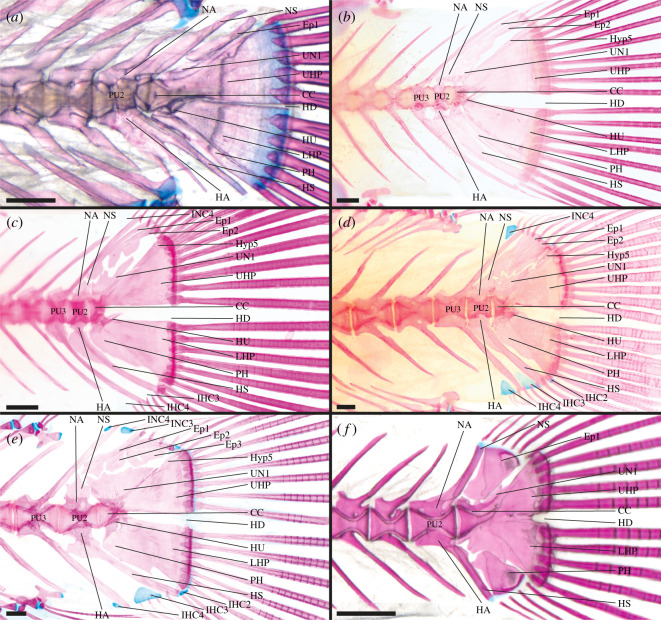
Caudal-fin skeleton of cleared and stained specimens of (*a*) Congrogadidae—*Halimuraena hexagonata* (MNHN 2020 0380, SL = 52.0 mm); (*b*) Plesiopidae—*Plesiops* sp. (DMM IE/13299, SL = 48.1 mm); (*c*) Grammatidae—*Gramma loreto* (DMM IE/16529, SL = 41.2 mm); (*d*) Pseudochromidae—*Pseudochromis aldabraensis* (DMM IE/10342, SL = 73.9 mm); (*e*) Opistognathidae—*Opistognathus aurifrons* (DMM IE/16518, SL = 72.1 mm); (*f*) Gobiesocidae—*Diplecogaster bimaculata* (MNHN uncat, SL = 33.5 mm). CC, compound centrum; HD, hypural diastema; Ep, epural; IHC, inter-haemal spine cartilage; INC, inter-neural spine cartilage; HA, haemal arch; HS, haemal spine; HU, hypurapophysis; Hyp, hypural; LHP, lower hypural plate; NA, neural arch; NS, neural spine; PH, parhypural; PU, preural centrum; UHP, upper hypural plate; UN, uroneural. Scale bar = 1 mm.

**Congrogadidae**—1 out of 8 genera examined, e.g. *Halimuraena hexagonata* (figure 6*a*).

CC and PU2 contribute to the caudal fin; components of the CC unknown [CC (?)]; LHP present, LHP articulates with CC; UHP present, UHP fused to CC, HD absent; presence of Hyp5 questionable (either part of UHP or absent); PH articulates with CC, fused to LHP; HU absent; UN present, UN fused to CC and UHP; one Ep present; HA of PU2 and HA of PU3 fused to respective centrum; NS of PU2 normal length; no IHC or INC present.

**Plesiopidae**—7 out of 12 genera examined [46,84,87,89,91], e.g. *Plesiops* sp. (Figure 6*b*).

CC, PU2 and PU3 contribute to the caudal fin; components of CC unknown [CC (?)]; LHP present, LHP articulates with CC; UHP present, UHP fused to CC; Hyp5 present, Hyp5 shortened, Hyp5 not fused to UHP nor to CC; PH articulates with CC, not fused to LHP; HU present, HU splint-like and directed postero-dorsally; UN present, UN fused to CC, not fused to Hyp5 or UHP; three Ep present; HA of PU2 articulates with centrum, HA of PU3 fused to centrum; NS of PU2 shortened; IHC2, IHC3 and IHC4 present, INC3 and INC4 present.

Hyp5 is severely shortened in *Acanthoplesiops psilogaster*, *A. hiatti* and *Trachinops noarlungae*. The PH is fused to the LHP in *Acanthoclinus*, *Acanthoplesiops*, *Belonepterygion fasciolatum*, *Beliops xanthokrossos* and *Steeneichthys plesiopsus*. The HU is elongated in *Plesiops coeruleolineatus* and *B. fasciolatum*, while it is short in *Acanthoclinus fuscus*, *Acanthoclinus littoreus* and *Beliops xanthokrossos* and completely absent in *A. psilogaster*. The UN is additionally fused to Hyp5 in *Acanthoclinus littoreus*, *B. fasciolatum*, *S. plesiopsus* and *T. noarlungae*. In *Plesiops* sp. (Figure 6*b*) and *S. plesiopsus* the two posterior epurals are fused. The HA of PU2 is fused to its centrum in *Acanthoclinus* sp., *Acanthoclinus fuscus*, *Acanthoplesiops*, *B. fasciolatum* and *Beliops xanthokrossos*. The NS of PU2 is present in its complete length in *Acanthoplesiops psilogaster* while in *T. noarlungae* this NS is short. In *S. plesiopsus* IHC2 and INC3 are missing and in *P. coeruleolineatus* IHC3 is absent (figure 6*b*).

**Grammatidae**—2 out of 2 genera examined, e.g. *Gramma loreto* (figure 6*c*).

CC, PU2 and PU3 contribute to the caudal fin; components of the CC unknown [CC (?)]; LHP present, LHP articulates with CC; UHP present, UHP fused to CC; Hyp5 present, Hyp5 not fused to UHP nor CC, Hyp5 shortened or severely shortened; PH articulates with CC, fused to LHP; HU present on PH, HU splint-like directed postero-dorsally; UN present, UN not fused to CC nor to UHP or fused to both; two Ep present; HA of PU2 and PU3 articulate with respective centrum; NS of PU2 truncated; IHC2, IHC3 and IHC4 present, INC4 present.

Recent molecular analyses suggest that the Grammatidae do not form a monophyletic taxon [2]. The two genera *Gramma* and *Lipogramma* are rather considered to be distinct taxa within the Ovalentaria. In the genus *Lipogramma* Hyp5 is severely shortened while in *Gramma* Hyp5 is short. By contrast, the UN in *Gramma* is fused to the CC and the UHP (figure 6*c*) while it is separated in *Lipogramma*.

**Pseudochromidae**—8 out of 16 genera examined [46,83], e.g. *Pseudochromis aldabraensis* (figure 6*d*).

CC, PU2 and PU3 contribute to the caudal fin; components of CC unknown [CC (?)]; LHP present, LHP articulates with CC; UHP present, UHP fused to CC; LHP and UHP contact each other anterior; Hyp5 present, Hyp5 not fused to UHP nor CC, Hyp5 severely shortened; PH articulates with CC, PH fused to LHP; HU present on PH; UN present, UN fused to CC and Hyp5; two Ep present; HA of PU2 and PU3 fused to respective centrum; NS of PU2 truncated, outgrowth of membrane bone dorsal to NA; IHC2, IHC3 and IHC4 present, (INC3) and INC4 present.

In *Labracinus* and *Ogilbyina queenslandiae* the PH is not fused to the LHP. In *Labracinus* the UN is only fused to the CC. Three epurals are present in *Chlidichthys johnvoelckeri*, *Labracinus*, *O. queenslandiae* and *Pectinochromis lubbocki*. Presence of INC3 in the ground plan of pseudochromids is uncertain because of its distribution within the different species. INC3 is present in some specimens of *Amsichthys knighti*, in *Labracinus*, *Lubbockichthys* sp., *P. lubbocki* and *Pseudoplesiops typus*.

**Opistognathidae**—3 out of 4 genera examined [90], e.g. *Opistognathus aurifrons* (figure 6*e*).

CC, PU2 and PU3 contribute to the caudal fin; components of CC unknown [CC (?)]; Hyp1 + Hyp2 fused to form LHP, LHP articulates with CC; Hyp3 + Hyp4 fused to form UHP, UHP fused to CC; Hyp5 present, Hyp5 severely shortened, Hyp5 not fused to UHP nor CC; PH articulates with CC, PH fused to LHP; HU present on PH, HU splint-like directed postero-dorsally; UN present, UN fused to CC, not fused to Hyp5 or UHP; three Ep present; HA of PU2 articulates with centrum; NS of PU2 truncated; IHC2, IHC3 and IHC4 present, INC3 and INC4 present.

In an examined juvenile specimen of *Stalix* sp. an elongated UC was observed, which is similar to the condition found in cichlids. We hypothesize that in opistognathids too, only one UC develops and forms the CC. The HU is absent in *Opistognathus aurifrons* (figure 6*e*) and *O. rosenblatti*. In *O. aurifrons* (figure 6*e*) and *O. rosenblatti* the HA of PU2 is fused to its centrum. In *O. darwiniensis* IHC4 and INC3 and INC4 are absent.

### 3.1.8. Gobiesociformes

**Gobiesocidae**—6 out of 53 genera examined [46,86,88,94], e.g. *Diplecogaster bimaculata* (figure 6*f*).

CC and PU2 contribute to the caudal fin; components of CC unknown [CC (?)]; LHP present, LHP fused to CC; UHP present, UHP fused to CC; LHP and UHP partially fused; presence of Hyp5 questionable (either part of UHP or absent); PH separate from CC, PH not fused to LHP; HU absent; UN present, UN fused to CC; one Ep present; HA of PU2 and PU3 fused to respective centrum; NS of PU2 normal length, IHC and INC absent.

Within the Gobiesocidae the size of the HD varies and may be dependent on specimen size. In a smaller specimen of *Apletodon dentatus* the HD is comparable to that of other ovalentarian taxa in terms of its size. In a larger specimen it is anteriorly restricted due to the fusion of the LHP and UHP. In *Diplecogaster bimaculata* (figure 6*f*) the HD is more anteriorly restricted, while in *Kopua minima* the HD is narrow due to the close location of LHP and UHP. In *Gobiesox strumosus*, *Gouania willdenowi* and *Lepadogaster* the HD are again anteriorly restricted and partly narrowed. Vaz & Hilton [94] showed no PH nor an Ep for *G. strumosus* (cartilaginous elements not stained), while Rosen & Patterson [88] reported no Ep for *G. funebris*. The UN is additionally fused to the UHP in *K. minima* and *G. strumosus* while no UN is present in *Gouania willdenowi*. In *L. lepadogaster* IHC3 is present and INC3 may be present.

### 3.1.9. Blenniiformes

**Figure 7.**
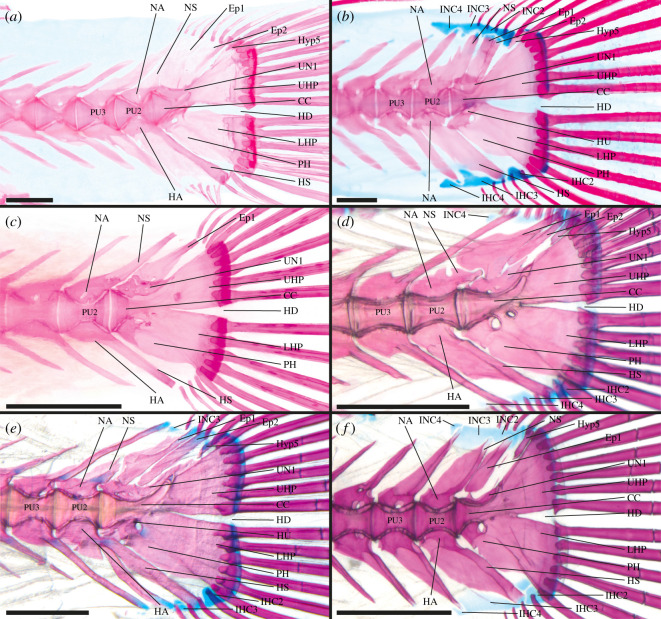
Caudal-fin skeleton of cleared and stained specimens of (*a*) Tripterygiidae—*Tripterygion delaisi* (DMM IE/12013, SL = 53.7 mm); (*b*) Blenniidae—*Lipophrys pholis* (DMM IE/11164, SL = 59.8 mm); (*c*) Clinidae—*Clinitrachus argentatus* (DMM IE/16705, SL = 26.9 mm); (*d*) Labrisomidae—*Malacoctenus delalandii* (MNHN 2020 0386, SL = 21.6 mm); (*e*) Labrisomidae—*Paraclinus altivelis* (MNHN 2020 0387, SL = 17.3 mm); (*f*) Chaenopsidae—*Emblemaria hypacanthus* (MNHN 2020 0381, SL = 25.0 mm). CC, compound centrum; HD, hypural diastema; Ep, epural; IHC, inter-haemal spine cartilage; INC, inter-neural spine cartilage; HA, haemal arch; HS, haemal spine; HU, hypurapophysis; Hyp, hypural; LHP, lower hypural plate; NA, neural arch; NS, neural spine; PH, parhypural; PU, preural centrum; UHP, upper hypural plate; UN, uroneural. Scale bar = 1 mm.

**Tripterygiidae**—28 out of 29 genera examined [46,85], e.g. *Tripterygion delaisi* (figure 7*a*).

CC, PU2 and PU3 contribute to the caudal fin; components of CC unknown [CC (?)]; LHP present, LHP articulates with CC; UHP present, UHP fused to CC; Hyp5 present, Hyp5 not fused to UHP nor to CC, Hyp5 severely shortened; PH articulates with CC, fused to LHP; HU present on PH, splint-like and directed postero-dorsally; UN present, UN fused to CC; two Ep present; HA of PU2 and PU3 fused to respective centrum; NS of PU2 normal length, IHC and INC absent.

In many tripterygiid species only the CC and PU2 contribute to the caudal fin (e.g. *Apopterygion oculus*, *Notoclinus fenestratus*). Further, the length of Hyp5 varies with *Norfolkia brachylepis* having a normal length Hyp5, many species (e.g. *Acanthanectes rufus*, *Apopterygion oculus*) having a shortened Hyp5 and *Helcogramma decurrens* missing Hyp5 completely. A UN is missing in some species (e.g. *Axoclinus lucillae*, *Notoclinops*). If present, the UN can additionally be fused to the UHP (e.g. *Enneanectes carminalis*, *Enneapterygius etheostoma*). The NS of PU2 may be short (e.g. *A. lucillae*, *Tripterygion delaisi*) or truncated (e.g. *Enneanectes reticulatus*, *Lepidonectes corallicola*). In *E. etheostoma* IHC3 and IHC4 are present. There are no IHC or INC reported for other tripterygiid species.

**Blenniidae**—13 out of 58 genera examined [46,91,94,95], e.g. *Lipophrys pholis* (figure 7*b*).

CC, PU2 and PU3 contribute to the caudal fin; CC presumably forms from one UC [CC (UC1/UC2?)]; LHP present, LHP articulates with CC; Hyp3 + Hyp4 fused together to form UHP, UHP fused to CC; Hyp5 present, Hyp5 not fused to UHP nor to CC, Hyp5 severely shortened; PH articulates with CC and LHP; HU present on PH, HU splint-like and directed postero-dorsally, HU shortened; UN present, UN fused to CC and UHP; three Ep present; HA of PU2 and PU3 fused to respective centrum; NS of PU2 normal length; IHC2 + IHC3 + IHC4 (connected) present, INC2 + INC3 + INC4 (connected) present.

Developmental data of *Enchelyurus brunneolus* suggest that only one UC develops during ontogeny [95]. It cannot be determined if this UC represents UC1, UC2 or if it is a product of evolutionary fusion of these two UC. In *Chasmodes bosquianus* the PH and LHP are fused to the CC. In *Aspidontus taeniatus* and *Plagiotremus tapeinosoma* the LHP has an outgrowth along the middle of its dorsal margin while the UHP has an outgrowth along the middle of its ventral margin which results in severely anteriorly restricted HD. In *Aspidontus taeniatus*, *Plagiotremus tapeinosoma* and *C. bosquianus* no Hyp5 is present. Watson [95] reported a small cartilage dorsal to the UHP in his developmental stages of *E. brunneolus* but interpreted it as a radial cartilage rather than Hyp5. Since Hyp5 is present in most other examined blenniids at the same position, it seems reasonable to interpret this structure to be Hyp5. Hyp5 is shortened in *Hypleurochilus geminatus*. In *C. bosquianus* and *E. brunneolus* the LHP and UHP are partially fused. In only *Ecsenius bicolor* three Ep are present; in other Blenniidae there are two (e.g. *Lipophrys*, *Omobranchus*) or one Ep (e.g. *A. taeniatus*, *C. bosquianus*) present. The NS of PU2 is short in *Istiblennius enosimae* and *Parablennius*. IHC5 is present in *Omobranchus elegans* and INC5 is present in *Omobranchus*.

**Clinidae**—6 out of 26 genera examined [82,96], e.g. *Clinitrachus argentatus* (figure 7*c*).

CC and PU2 contribute to the caudal fin; CC forms from one UC [CC (UC1/UC2?)]; Hyp1 + Hyp2 fused to form LHP, LHP fused to CC; Hyp3 + Hyp4 fused to form UHP, UHP fused to CC; HD narrowed; Hyp5 present, Hyp5 severely shortened, Hyp5 not fused to UHP nor to CC; PH fused to CC and LHP; HU absent; UN present, UN fused to CC and UHP; two Ep present; HA of PU2 and PU3 fused to respective centrum; NS of PU2 normal length; IHC2 present, INC absent.

The developmental data of *Clinus cottoides* and *Myxodes viridis* suggest that only one UC develops during ontogeny and is eventually reduced in size to form the CC [82,96]. It cannot be determined if this UC represents UC1, UC2 or if it is a product of evolutionary fusion of these two UC. Due to the fusion of the LHP and UHP, the diastema is narrowed in all clinids and is small in *Heteroclinus heptaeolus* and *Ericentrus rubrus*. The LHP and UHP are partially fused in *Clinus*, *Cristiceps*, *Heteroclinus* and *Ericentrus*. While Hyp5 is present in some *Heteroclinus* species, Hyp5 is not distinguishable in the other examined species and developmental data of *Myxodes viridis* indicate that no Hyp5 is developed in this species. Only one Ep is present in *Clinitrachus argentatus*. IHC3 was only observable in *H. perspicillatus* and *H. tristis*.

**Labrisomidae**—5 out of 16 genera examined, e.g. *Malacoctenus delalandii* (figure 7*d*) and *Paraclinus altivelis* (figure 7*e*).

CC, PU2 and PU3 contribute to the caudal fin; CC presumably forms from one UC [CC (UC1/UC2?)]; Hyp1 + Hyp2 fused to form LHP, LHP fused to CC; Hyp3 + Hyp4 fused to form UHP, UHP fused to CC; Hyp5 present, Hyp5 not fused to UHP nor to CC, Hyp5 severely shortened; PH articulates with CC, fused to LHP; HU present on PH, HU very small and directed laterally; UN present, UN fused to CC and UHP; two Ep present; HA of PU2 and PU3 fused to respective centrum; NS of PU2 of truncated; IHC2, IHC3 and IHC4 present, INC3 and INC4 present.

The most recent molecular analyses suggest that the Labrisomidae do not form a monophyletic taxon [2,108]. A juvenile specimen of *Paraclinus marmoratus* indicates that only one UC is developed and forms the CC as it very much resembles the developmental shape of the CC in blenniids. It cannot be determined if this UC represents UC1, UC2 or if it is a product of evolutionary fusion of these two UC. In *Dialommus fuscus*, *Gobioclinus dendriticus*, *Malacoctenus* and *Labrisomus nuchipinnis* the HU is absent. In *P. altivelis* INC4 is missing (figure 7*e*).

**Chaenopsidae**—4 out of 13 genera examined [46], e.g. *Emblemaria hypacanthus* (figure 7*f*).

CC, PU2 and PU3 contribute to the caudal fin; components of the CC unknown [CC (?)]; LHP present, LHP fused to CC; UHP present, UHP fused to CC; LHP and UHP partially fused; Hyp5 present, Hyp5 severely shortened, Hyp5 not fused to UHP; PH fused to CC, PH fused to LHP; HU absent; UN present, UN fused to CC; two Ep present; HA of PU2 and PU3 fused to respective centrum; NS of PU2 normal length; IHC2 and IHC3 + IHC4 (connected) present, INC2 + INC3 + INC4 (connected) present.

Only the CC and PU2 contribute to the caudal fin of *Mccoskerichthys sandae*. In *Neoclinus bryope* the LHP and PH articulate with the CC. Also, the LHP and UHP are not fused. A very small HU is present in *N. bryope*. In *M. sandae* Hyp5 is not distinguishable, but it seems likely that it is part of the UHP as this plate is larger in *M. sandae* than in the other examined species and occupies the space where Hyp5 is expected to be. Only one Ep is present in *Emblemaria hypacanthus* and *M. sandae*. There are no IHC4 and INC4 present in *M. sandae*.

**Dactyloscopidae**—5 out of 9 genera examined [80,81,94].

CC and PU2 contribute to the caudal fin; components of CC unknown [CC (?)]; LHP present, LHP fused to CC; UHP present, UHP fused to CC; LHP and UHP partly fused anteriorly, HD anteriorly restricted; Hyp5 not distinguishable (either fused to UHP or absent); PH fused to CC and to LHP; HU absent; UN present, UN fused to CC and to UHP; two Ep present; HA of PU2 and PU3 fused to respective centrum; NS of PU2 normal length; IHC2 present, INC2 present.

While the UN is easily distinguished in *Gillellus semicinctus* and *Leurochilus acon*, its dorsal portion is very much reduced in the other examined species. In *Dactylagnus mundus* and *Gillellus semicinctus* only one Ep is present.

### 3.2. Ancestral character state reconstruction

**Figure 8.**
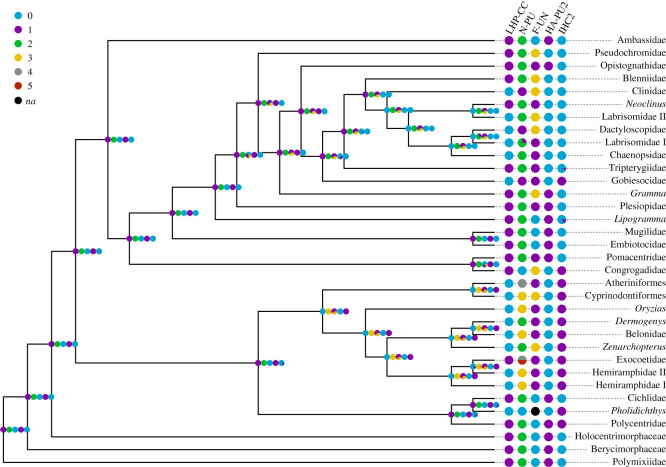
Results from the ancestral character state reconstruction of five selected characters plotted on the topology provided by Betancur-R *et al*. [2]. States of each character are shown at the tips of the tree for each taxon. Probability of each character state for the respective character are plotted at each node. LHP-CC, fusion of lower hypural plate with compound centrum: 0—fused, 1—unfused; N-PU, number of preural centra; F-UN, fusion of uroneural: 0—unfused, 1—fused to compound centrum, 2—fused to upper hypural, 3—fused to compound centrum and upper hypural; HA-PU2, fusion of haemal arch to centrum of preural centrum 2: 0—fused, 1—unfused; IHC2, presence of inter-haemal cartilage 2: 0—present, 1—absent.

The ancestral character state reconstructions (figure 8 and electronic supplementary material, S4) based on the phylogenetic tree provided by Betancur-R *et al*. [2] revealed several characters that support the Atherinomorpha as a clade as well as the clade including *Lipogramma* up to the Blenniimorphae (*sensu* [2]), or subgroups thereof. The Atherinomorpha are supported by five characters likely present in their last common ancestor: (i) three preural centra support the caudal fin, (ii) fusion of the LHP with the CC, (iii) fusion of UN with the CC, (iv) fusion of the HA of PU2 with the respective centrum and (v) absence of IHC2. The taxon including Grammatidae, Plesiopidae, Pseudochromidae, Opistognathidae and the Blenniimorphae are supported by a severely shortened Hyp5 and the fusion of the PH with the LHP. *Gramma* and the Blenniimorphae share the fusion of UN with the CC. The analysis indicates that this fusion may have occurred early in evolution, either before the split of the Pseudochromidae or even before the split of the Plesiopidae. Furthermore, the most-recent common ancestor of the Blenniimorphae shares the fusion of the HA of PU2 with its respective centrum, which appears to have evolved convergently in Atherinomorpha. The common ancestor of the taxa Chaenopsidae, Clinidae, Dactyloscopidae and Labrisomidae supposedly had the HU missing, and the LHP fused to the CC, the latter seemingly evolved independently in Atherinomorpha.

The ancestral character state reconstruction also provides a possible ground plan for the most-recent common ancestor of all ovalentarian taxa. However, an explicit state cannot be identified for all characters. This most-recent common ancestor probably had a forked caudal fin supported by two preural centra. The lower hypurals were fused and formed a LHP while hypurals 2 and 3 and hypurals 4 and 5 were separate. The PH and the LHP articulated with the CC. A full-length hypural 5 was present. A diastema separated the LHP from the upper hypurals. One UN was present, which was not fused to the CC or the upper hypurals. The HA of preural centrum 2 was not fused to the respective centrum but the HA of preural centrum 3 was. The NS of preural centrum 2 was very short. Two epurals were present just as inter-haemal cartilages 2 and 3.

### 3.3. Phylogenetic analysis

**Figure 9.**
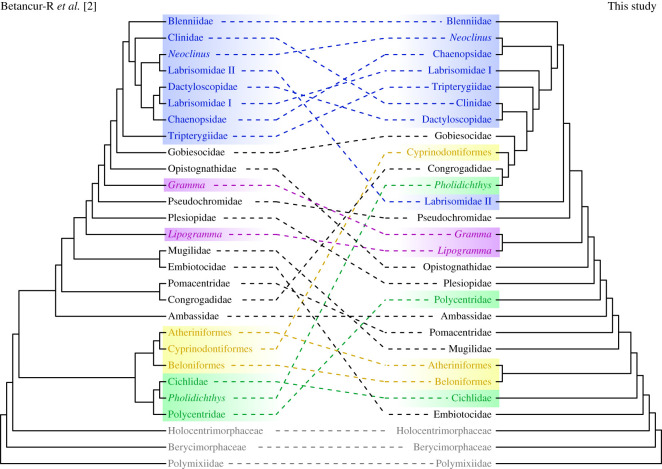
Comparison of phylogenetic relationships of ovalentarian taxa based on molecular data (Betancur-R *et al*. [2]) and morphological data (this study, Bayesian inference). Green, ‘Cichlidae + Polycentridae + *Pholidichthys*'; yellow, Atherinomorpha; violet, Grammatidae; blue, Blenniiformes.

The results of the different phylogenetic analyses provide very similar phylogenetic hypotheses representing the evolution of the caudal-fin skeleton within the Ovalentaria. The earliest branching taxon within the Ovalentaria was either the Embiotocidae (BI, figure 9, supporting character state changes listed in electronic supplementary material, S6) or the Cichlidae (MP and ML, electronic supplementary material, S5). Atheriniformes and Beloniformes were retrieved as sister taxa; however, the Cyprinodontiformes are resolved as more derived. Together with the Gobiesocidae, Congrogadidae and Pholidichthyidae, Cyprinodontiformes are the most derived taxa retrieved from the analyses. The Mugilidae are retrieved as closely related to the Atheriniformes/Beloniformes (BI; figure 9) or even as their sister taxon (MP and ML; electronic supplementary material, S5). In all analyses, pomacentrids, polycentrids, ambassids, plesiopids and opistognathids are more derived than the mugilids. *Gramma* and *Lipogramma* are retrieved as sister taxa. The Pseudochromidae are positioned as sister taxon to the Blenniiformes including the Gobiesocidae, Cyprinodontiformes, Congrogadidae and Pholidichthyidae. Relationships within the Blenniiformes vary among the analyses (electronic supplementary material, S5).

Although the support values provided by these analyses are only moderate, this can be attributed to the rather small set of characters used.

## 4. Discussion

### 4.1. Evolutionary trends

The wide array of caudal-fin shapes that can be found in ovalentarian taxa (figure 1) is surpassed by the different compositions of the caudal-fin skeletons in this clade. Many authors presented detailed descriptions of caudal fins for numerous ovalentarian taxa (e.g. [46]), but none of them compared these taxa in a phylogenetic context. When viewed in a phylogenetic context, the caudal-fin skeletons revealed different evolutionary trends that are observable within the Ovalentaria.

(1) Reduction of the overall number of hypural elements. First, hypurals 1 and 2 fuse to form a LHP. While in few ovalentarian taxa, i.e. Cichlidae, Polycentridae and Pomacentridae, these two elements remain separate, they are fused in all other taxa. Developmental data suggest that the tendency of fusion is reflected by the time it occurs during ontogeny. While in more basal ovalentarian taxa, e.g. mugilids, atheriniforms and beloniforms, the individual hypurals are still preformed as separate cartilages and then fuse [68,70,93], in more derived taxa, e.g. blenniids and clinids, the LHP already develops from a single cartilaginous element [82,133]. Also, within the Pomacentridae the fusion of the lower hypurals evolved independently, emphasizing the tendency of the reduction of the amount of hypural elements [46]. Second, a similar trend is observable for hypurals 3 and 4, which are also fused in the majority of ovalentarian taxa. While these elements remain separate in atheriniforms, cichlids, embiotocids, polycentrids and pomacentrids, this supports the hypothesis that hypurals tend to fuse, as it requires several independent acquisitions of this feature. Third, the fifth hypural is reduced in size in several ovalentarian taxa (e.g. Blenniidae, Labrisomidae, Tripterygiidae) and finally absent in others (e.g. Congrogadidae, Cyprinodontiformes, Dactyloscopidae and Gobiesocidae). A shortened hypural 5 is found in many taxa, but within the taxon assemblage that includes grammatids, pseudochromids, gobiesocids and blenniiforms, it is severely shortened and missing in two of the included families (dactyloscopids and gobiesocids). The overall reduction of the number of hypural elements results in less flexible and stiffer hypural plates. This is further emphasized by the fusion of the lower and UHP and the fusion of the PH to the LHP in some taxa.
(2) Fusion of the hypurals to the CC. Both the lower hypurals and the upper hypurals, or their respective plates, tend to fuse to the CC. Fusion of the LHP with the CC is present in atherinomorphs and the Blenniiformes (except for the blenniids) as well as pholidichthyids and gobiesocids (figure 8). The fusion of the upper hypurals with the CC is present in almost all ovalentarian taxa except atheriniforms, cichlids, embiotocids and the beloniform families Belonidae, Exocoetidae and Hemiramphidae. The fusion of the hypural elements with the CC results in a stiffened caudal-fin complex.
(3) Transition from a forked fin shape to a rounded fin shape (figure 1). Only few taxa within the Ovalentaria retain a forked caudal fin, i.e. Ambassidae, Atheriniformes, Beloniformes, Embiotocidae, Mugilidae and Pomacentridae. Within the Atheriniformes and Beloniformes many species have altered caudal-fin shapes, e.g. many melanotaeniids have lunate caudal fins and zenarchopterids have rounded caudal fins.

The illustrated evolutionary trends correlate with the locomotion types employed by the different ovalentarian taxa. While the locomotion of fishes is well investigated [12,17,18,22] (e.g. [23,24,25,134]), the influence of the caudal-fin skeleton on locomotion and vice versa was only considered by Gosline [26]. Herein we shortly want to introduce two examples on the likely interaction between caudal-fin skeleton and the locomotion of the respective taxa.

Mugilids use a combination of BCF propulsion and MPF propulsion [22–24]. Such a combination gives them fairly good cruising and accelerating abilities but still allows for good manoeuvrability [23]. Further, for their BCF propulsion mugilids use a carangiform motion [22]. Required for such a locomotion is a certain degree of movability of the skeletal elements of the caudal fin and at the same time some stiffness in the caudal-fin skeleton as the generated forward forces need to be counteracted [26]. In mugilids this is achieved by the fusion of hypurals 1 and 2 as well as hypurals 3 and 4 that are also fused to the CC. This creates a moderate stiffness in the caudal fin but still allows for a lateral movement of the lower and upper fin lobes, which externally is reflected by the forked shape of the caudal fin.

Blenniids are more specialized in their locomotion. In their bottom-dwelling lifestyle, they mainly use MPF propulsion, and their caudal fin is primarily used for manoeuvring. The caudal fin serves as an elongation of the body that not only generates an undulatory motion but supports the undulatory movement of the median fins. The rounded shape of the caudal fin fits this type of locomotion as upper and lower fin lobes are reduced [24]. Further, the caudal-fin skeleton needs neither flexible elements which allow for a high degree of independent movement of the lower and upper fin elements nor stiffened elements to counteract strong forces. However, in blenniids single skeletal elements become reduced by fusion, i.e. hypurals 1 and 2, hypurals 3 and 4, UHP to CC or are reduced in size, i.e. hypural 5 (figure 8*a*). This seems necessary as such a reduction stiffens the caudal peduncle, reduces independent movements of the caudal fin, and, therefore, results in a direct prolongation of the horizontal axis of the vertebral column up to the posterior tip of the caudal fin.

The influence of the caudal-fin skeleton on the mode of locomotion and vice versa obviously is not well studied. The two examples discussed above emphasize the missed opportunities in not examining the skeleton when analysing the locomotion of fishes. Combining these two fields of study can give new insights into the evolution of modes of locomotion within different teleost taxa and simultaneously reveal associated changes in the caudal-fin skeleton.

### 4.2. Ground plan of the ovalentarian caudal-fin skeleton

The reconstruction of the ground plan of the Ovalentaria provides an overview of possible character states in the most-recent common ancestor of ovalentarian taxa. In general, the states of most characters (e.g. number of preural centra contributing to the caudal fin, single upper hypurals, connection of hypurals to CC) are similar to that of the chosen outgroups (*Polymixia*, Berycimorphaceae and Holocentridae). However, a few character states seem questionable although their reconstructed probabilities are unambiguous, e.g. fusion of hypural 1 and hypural 2. Regarding this specific character, one would assume that these two elements were separate in the most-recent common ancestor of all ovalentarian taxa as they remain separate in some of the basal most taxa, i.e. Cichlidae, Embiotocidae, Polycentridae, Pomacentridae, as well as in the outgroup taxa. Furthermore, developmental data show that even in some of the more basal taxa, in which these hypurals are fused in adults, they develop separately during ontogeny [33,68,70,93,130,135]. However, the underlying phylogenetic hypothesis has very low support values for the basal nodes within the Ovalentaria [2]. Therefore, the topology of the phylogenetic tree can be questioned. For the described examples a slightly altered topology might change the results of the character state reconstruction at the base of the Ovalentaria. For other characters, the topology of basal ovalentarian taxa seems to have less impact.

### 4.3. Phylogenetic relationships of ovalentarian taxa

This is the first phylogenetic analysis of morphological characters that includes all ovalentarian taxa. The taxon assemblage proposed by molecular data includes between 42 and 48 families, depending on author [19,55], which previously were widely scattered within the Percomorpha. Hence, no study has analysed morphological data for this specific composition of taxa.

Phylogenetic reconstruction based on characters from the caudal-fin skeleton provides phylogenetic scenarios for the evolution of the caudal-fin skeleton within the Ovalentaria. Because the phylogenetic relationships retrieved in our analyses are based solely on one character complex, a certain influence of ecological as well as functional factors is likely, as seen in the derived positions of gobiesocids, cyprinodontiforms, congrogadids and pholidichthyids. This differs greatly from the phylogenetic positions of these taxa retrieved from molecular data [1,2,6,54]. Due to their highly modified caudal-fin skeleton, e.g. reduced number of hypurals, hypural plates fused to the CC, which is much like that of dactyloscopids or clinids, they are positioned within the Blenniiformes (*sensu* [2]). These morphological congruences may be attributed to similar demands towards their locomotion in their respective habitats and therefore can be regarded as convergencies. The phylogenetic signal of the caudal-fin skeleton characters of these taxa may be overshadowed by adaptations to similar habitats. Especially the retrieved position of cyprinodontiforms is questionable as both morphological and molecular analyses previously supported the close relationship of atheriniforms, beloniforms and cyprinodontiforms [1,2,52,54,57,132].

Nonetheless, there are congruences between the herein retrieved phylogenetic relationships of ovalentarian taxa and recent phylogenetic hypothesis based on molecular data (figure 9) [2,6,54]. An important similarity between molecular and morphological phylogenetic analyses is the position of atheriniforms/beloniforms and cichlids at the base of the Ovalentaria (figure 9). Polycentrids and pholidichthyids, which are more closely related to cichlids according to molecular analyses, are spread along the phylogenetic tree based on morphological data. The Blenniiformes (*sensu* [2]) were also retrieved by morphological data; however, their intra relationships differ (figure 9).

In the most recent molecular-based phylogenetic hypothesis, the position of many taxa, e.g. Ambassidae, Embiotocidae, Mugilidae and Pomacentridae, remained uncertain due to low support values [2]. Morphological analyses now provide a different hypothesis for their relationships (figure 9; electronic supplementary material, S5). Embiotocids are recovered as the earliest branching taxon with the Ovalentaria. Mugilids are closely related to atheriniforms/beloniforms (sister taxa based on ML analysis; electronic supplementary material, S5). Such a relationship was previously supported by both morphological [57,132] and molecular data [136]. A closer relationship of ambassids and mugilids as proposed by Wainwright *et al*. [6] and Hughes *et al*. [54] cannot be fully ruled out from morphological phylogenetic analysis. However, Ambassidae, Mugilidae and Pomacentridae seem to be closer related which matches results from molecular phylogenetic analyses (figure 9). Further taxa, i.e. Plesiopidae, Pseudochromidae, Opistognathidae and Grammatidae, were retrieved in similar positions within both molecular-based and morphology-based analyses. While molecular data suggest that the two grammatid genera *Gramma* and *Lipogramma* are only distantly related, which results in paraphyletic Grammatidae [1,2], the morphological data support monophyletic Grammatidae (figure 9).

## 5. Conclusion

The present study shows that recent molecular phylogenies can contribute to new hypotheses in the evolution of morphological structures and morphological data can be used to independently test molecular findings. Both approaches work well together and can lead to new insights into the evolution of fish diversity. The phylogenetic analyses of the Ovalentaria using a dataset of one morphological complex, the caudal-fin skeleton, resulted in similar topologies as proposed by molecular data, which simultaneously support molecular findings and also demonstrate the power of morphological data. The well-known disadvantage, the time-consuming data acquisition, of morphological analyses should not be considered an obstacle as the results from such analyses bring forth new hypotheses useful in various disciplines, e.g. anatomy, evo–devo, functional morphology, phylogenetics, among many others.

## Data accessibility

Data and code supporting this paper are available as electronic supplementary material [137].
